# Review of guidance papers on regression modeling in statistical series of medical journals

**DOI:** 10.1371/journal.pone.0262918

**Published:** 2022-01-24

**Authors:** Christine Wallisch, Paul Bach, Lorena Hafermann, Nadja Klein, Willi Sauerbrei, Ewout W. Steyerberg, Georg Heinze, Geraldine Rauch, on behalf of topic group 2 of the STRATOS initiative

**Affiliations:** 1 Institute of Biometry and Clinical Epidemiology, Corporate Member of Freie Universität Berlin and Humboldt-Universität zu Berlin, Charité—Universitätsmedizin Berlin, Berlin, Germany; 2 Center for Medical Statistics, Informatics and Intelligent Systems, Section for Clinical Biometrics, Medical University of Vienna, Vienna, Austria; 3 School of Business and Economics, Emmy Noether Group in Statistics and Data Science, Humboldt-Universität zu Berlin, Berlin, Germany; 4 Faculty of Medicine and Medical Center, Institute of Medical Biometry and Statistics, University of Freiburg, Freiburg, Germany; 5 Department of Biomedical Data Sciences, Leiden University Medical Center, Leiden, The Netherlands; Witten/Herdecke University, GERMANY

## Abstract

Although regression models play a central role in the analysis of medical research projects, there still exist many misconceptions on various aspects of modeling leading to faulty analyses. Indeed, the rapidly developing statistical methodology and its recent advances in regression modeling do not seem to be adequately reflected in many medical publications. This problem of knowledge transfer from statistical research to application was identified by some medical journals, which have published series of statistical tutorials and (shorter) papers mainly addressing medical researchers. The aim of this review was to assess the current level of knowledge with regard to regression modeling contained in such statistical papers. We searched for target series by a request to international statistical experts. We identified 23 series including 57 topic-relevant articles. Within each article, two independent raters analyzed the content by investigating 44 predefined aspects on regression modeling. We assessed to what extent the aspects were explained and if examples, software advices, and recommendations for or against specific methods were given. Most series (21/23) included at least one article on multivariable regression. Logistic regression was the most frequently described regression type (19/23), followed by linear regression (18/23), Cox regression and survival models (12/23) and Poisson regression (3/23). Most general aspects on regression modeling, e.g. model assumptions, reporting and interpretation of regression results, were covered. We did not find many misconceptions or misleading recommendations, but we identified relevant gaps, in particular with respect to addressing nonlinear effects of continuous predictors, model specification and variable selection. Specific recommendations on software were rarely given. Statistical guidance should be developed for nonlinear effects, model specification and variable selection to better support medical researchers who perform or interpret regression analyses.

## Introduction

### Rationale

Knowledge transfer from the rapidly growing body of methodological research in statistics to application in medical research does not always work as it should [1]. Possible reasons for this problem are the lack of guidance and that not all statistical analyses are conducted by statistical experts but often by medical researchers who may or may not have a solid statistical background. Applied researchers cannot be aware of all statistical pitfalls and the most recent developments in statistical methodology. Keeping up is already challenging for a professional biostatistical researcher, who is often restricted to an area of main interest. Moreover, articles on statistical methodology are often written in a rather technical style making knowledge transfer even more difficult. Therefore, there is a need for statistical guidance documents and tutorials written in more informal language, explaining difficult concepts intuitively and with illustrative educative examples. The international STRengthening Analytical Thinking for Observational Studies (STRATOS) initiative (http://stratos-initiative.org) aims to provide accessible and accurate guidance documents for relevant topics in the design and analysis of observational studies [1]. Guidance is intended for applied statisticians and other medical researchers with varying levels of statistical education, experience and interest. Some medical journals are aware of this situation and regularly publish isolated statistical tutorials and shorter articles or even whole series of articles with the intention to provide some methodological guidance to their readership. Such articles and series can have a high visibility among medical researchers. Although some of the articles are short notes or rather introductory texts, we will use the phrase ‘statistical tutorial’ for all articles in our review.

Regression modeling plays a central role in the analysis of many medical studies, in particular, of observational studies. More specifically, regression model building involves aspects such as selection of a model type that matches the type of outcome variable, selection of explanatory variables to include in a model, choosing an adequate coding of the variables, deciding on how flexibly the association of continuous variables with the outcome should be modeled, planning and performing model diagnostics, model validation and model revision, reporting of a model and describing how well differences in the outcome can be explained by differences in the covariates. Some of the choices made during model building will strongly depend on the aim of modeling. Shmueli (2010) [2] distinguished between three conceptual modeling approaches: descriptive, predictive and explanatory modeling. In practice these aims are still often not well clarified, leading to confusion about which specific approach is useful in a modeling problem at hand. This confusion, and an ever-growing body of literature in regression modeling may explain why a common state-of-the-art is still difficult to define [3]. However, not all studies require an analysis with the most advanced techniques and there is the need for guidance for researchers without a strong background in statistical methodology, who might be “medical students or residents, or epidemiologists who completed only a few basic courses in applied statistics” according to the definition of level-1 researchers by the STRATOS initiative [1].

If suitable guidance for level-1 researchers in peer-reviewed journals was available, many misconceptions about regression model building could be avoided [4–6]. The researchers need to be informed about methods that are easily implemented, and they need to know about strengths and weaknesses of common approaches [3]. Suitable guidance should also point to possible pitfalls, elaborate on dos and don’ts in regression analyses, and provide software recommendations and understandable code for different methods and aspects. In this review, we focused on low-dimensional regression models where the sample size exceeds the number of candidate predictors. Moreover, we will not specifically address the field of causal inference, which goes beyond classical regression modeling.

So far, it is unclear what aspects of regression modeling have already been well-covered by related tutorials and where gaps still exist. Furthermore, suitable tutorial papers may be published but they are unknown to (nearly all) clinicians and therefore widely ignored in their analyses.

### Objectives

The objective of this review was to provide an evidence-based information basis assessing the extent to which regression modeling has been covered by series of statistical tutorials published in medical journals. Specifically, we sought to define a catalogue of important aspects on regression modeling, to identify series of statistical tutorials in medical journals, and to evaluate which aspects were treated in the identified articles and at which level of sophistication. Thereby, we put an intended focus on the choice of the regression model type, on variable selection and for continuous variables on the functional form. Furthermore, this paper will provide an overview, which helps to inform a broad audience of medical researchers about the availability of suitable papers written in English.

The remainder of this review is organized as follows: In the next section, the review protocol is described. Subsequently, we summarize the results of the review by means of descriptive measures. Finally, we discuss implications of our results suggesting potential topics for future tutorials or entire series.

## Material and methods

The protocol of this review describing the detailed design was already published by Bach et al. (2020) [7]. In here, we summarize its main characteristics.

### Eligibility criteria

First, we identified series of statistical tutorials and papers published in medical journals with a target audience mainly or exclusively consisting of medical researchers or practitioners. Second, we searched for topic-relevant articles on regression modeling within these series. Journals with a target audience of pure theoretical, methodological or statistical focus were not considered. We included medical journals if they were available in English language since this implies high international impact and broad visibility. Moreover, the series had to comprise at least five or more articles including at least one topic-relevant article. We focused on statistical series only since we believed that entire series have higher impact and visibility than isolated articles.

### Sources of information & search strategy

After conducting a pilot study for a systematic search for series of statistical tutorials, we had to adapt our search strategy since sensitive keywords to identify statistical series could not be found. Therefore, we consulted more than 20 members of the STRATOS initiative via email in spring 2018 for suggestions on statistical series addressing medical researchers. We also asked them to forward this request to colleagues, which resembles snowball sampling [8, 9]. This call was repeated at two international STRATOS meetings in summer 2018 and in 2019. The search was closed on June 30^st^, 2019. Our approach also included elements of respondent-driven sampling [10] by offering collaboration and co-authorship in case of relevant contribution to the review. In addition, we included several series that were additionally proposed by a reviewer during the peer-review process of this manuscript, and which were published by the end of June, 2019 to be consistent with the original request.

### Data management & selection process

The list of all resulting statistical series suggested is available as S1 File.

Two independent raters selected relevant statistical series from the pool of candidate series by applying the inclusion criteria outlined above.

An article within a series was considered to be topic-relevant if the title included one of the following keywords: *regression*, *linear*, *logistic*, *Cox*, *survival*, *Poisson*, *multivariable*, *multivariate*, or if the title suggested that the main topic of the article was *statistical regression modeling*. Both raters decided on the topic-relevance of an article independently and resolved discrepancies by discussion. To facilitate the selection of relevant statistical series, we designed a report form called *inclusion form* (S2 File).

### Data collection process

After the identification of relevant series and topic-relevant articles, a content analysis was performed on all topic-relevant articles using an *article content form* (S3 File). The article content form was filled-in for every identified topic-relevant article by the two raters independently and again discrepancies were resolved by discussion. The results of completed article content forms were copied into a data base for further quantitative analysis.

### Data items

In total 44 aspects of regression modeling were examined in the article content form (S3 File), which were related to four areas: *type of regression model*, *general aspects of regression modeling*, *functional form of continuous predictors*, and *selection of variables*. The 44 aspects cover topics of different complexity. Some aspects can be considered basic, others are more advanced. This was also commented in the S3 File for orientation. We mainly focused on predictive and descriptive models and did not consider particular aspects attributed to ethological models.

For each aspect, we evaluated whether it was mentioned at all, and if yes, the extent of explanation (short = one sentence only / medium = more than one sentence to one paragraph / long = more than one paragraph) [7]. We recorded whether examples and software commands were provided, and if recommendations or warnings were given with respect to each aspect. A box for comments provided space to note recommendations, warnings and other issues. In the article content form, it was also possible to add further aspects to each area. A manual for raters was created to support an objective evaluation of the aspects (S4 File).

### Summary measures & synthesis of results

This review was designed as an explorative study and uses descriptive statistics to summarize results. We calculated absolute and relative frequencies to analyze the 44 statistical aspects. We used stacked bar charts to describe the ordinal variable *extent of explanation* for each aspect. To structure the analysis, we grouped the aspects into the afore mentioned areas: *type of regression model*, *general aspects of regression modeling*, *determination of functional form for continuous predictors* and *selection of variables*.

We conducted the above analyses both article-wise and series-wise. In the article-wise analysis, each article was considered individually. For the series-wise analysis, the results from all articles in a series were pooled and each series was considered as the unit of observation. This means, if an aspect was explained in at least one article, this also counted for the entire series.

### Risk of bias

The risk of bias by missing a series was addressed extensively in the protocol of this study [7, 11, 12]. Moreover, bias could result from the inclusion criterion of series, which was the requirement of at least five articles in a series. This may have led to a less representative set of series. We set this inclusion criterion to identify highly visible series. Bias could also result from the specific choice of aspects of regression modeling to be screened. We tried to minimize this bias by the possibility for free text entries that could later be combined into additional aspects.

This review has been written according to the PRISMA reporting guideline [13, 14], compare S1 Checklist. This review does not include patients or humans. The data that were collected within the review are available in S1 Data.

## Results

### Selection of series and articles

**Fig 1:**
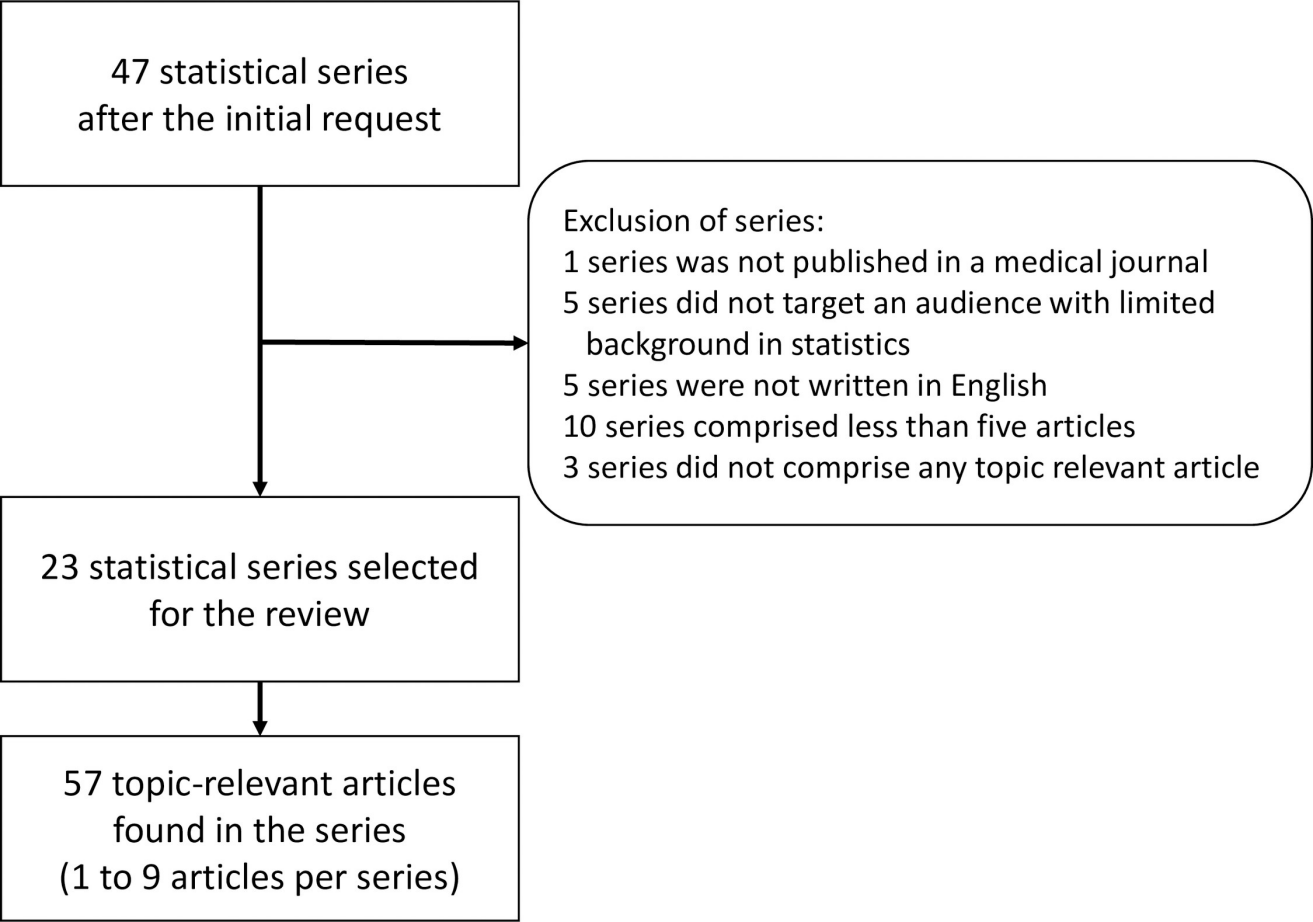
Flowchart of selection of statistical series and topic-relevant articles.

The initial query revealed 47 series of statistical tutorials (Fig 1 and S1 File). Out of these 47 series, two series were not published in a medical journal and five series did not target an audience with low statistical knowledge. Therefore, these series were excluded. Five and ten series were excluded because they were not written in English or they did not comprise at least five articles, respectively. Further, we excluded three series because they did not contain any topic-relevant article. The list of the series and the reason for each excluded series is found in S1 File. Finally, we included 23 series with 57 topic-relevant articles.

### Characteristics of the series

**Fig 2:**
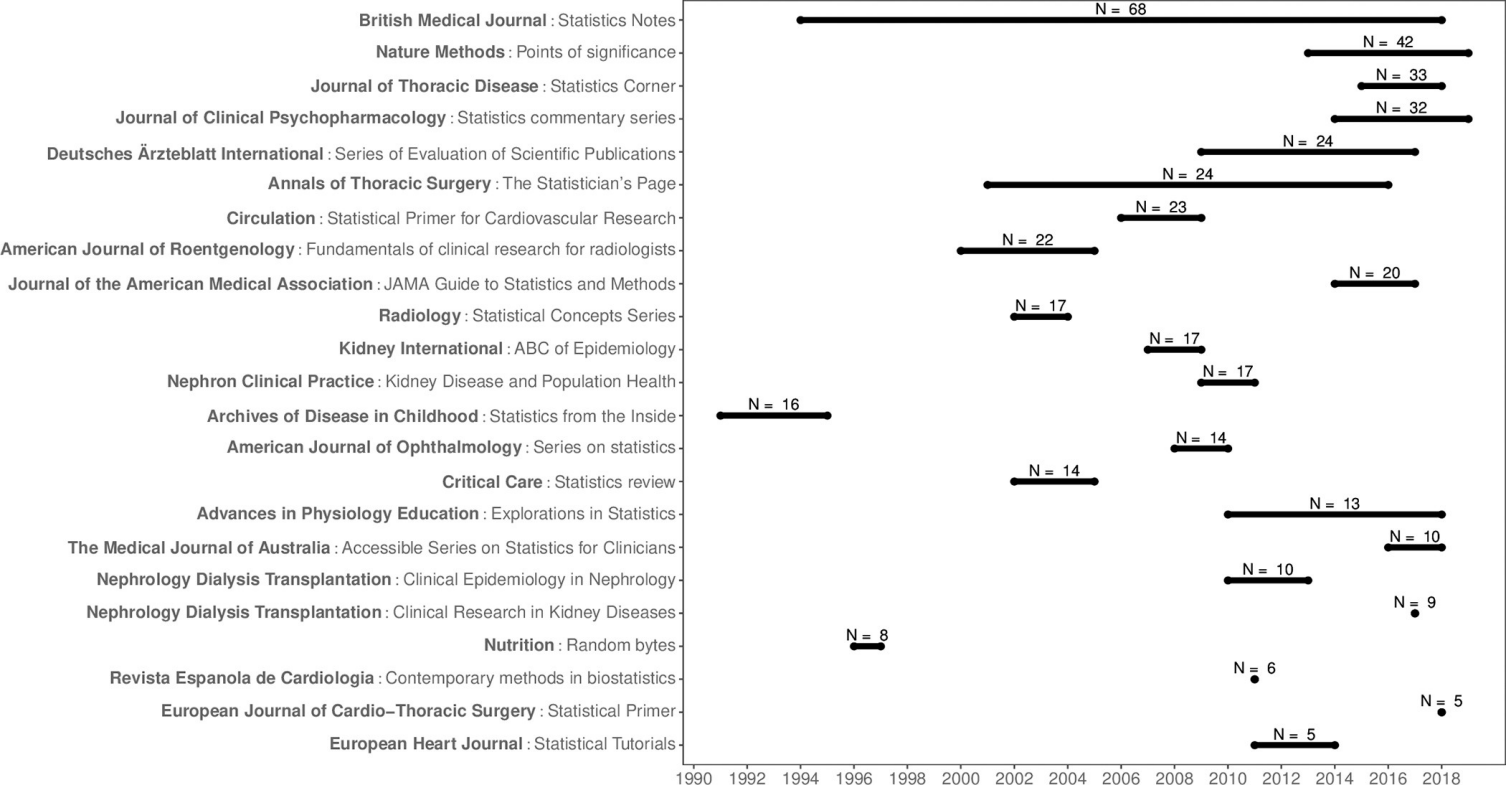
Publication years and number of articles in statistical series from highest to lowest.

Each series contained between one to nine topic-relevant articles (two on average, Table 1). The variability of the average number of article pages per series illustrates that the extent of the articles was very different (1 to 10.3 pages). Whereas the series *Statistics Notes* in the BMJ typically used a single page to discuss a topic, hence pointing only to the most relevant issues, there were longer papers with a length of up to 16 pages [15, 16]. The series in the *BMJ* is also the one spanning over the longest time period (1994–2018). Beside of the series in the *BMJ*, only the *Archives of Disease in Childhood* and the *Nutrition* series started publishing papers already in the last century. Fig 2 shows that most series were published only during a short period, perhaps paralleling terms of office of an Editor.

**Table 1:** Characteristics of included statistical series ranked by number of covered aspects. We considered 44 aspects, see S3 File.

Rank	Journal	Statistical series	Years of publications	No. of articles	No. of topic-relevant articles	Average no. of pages in topic-relevant articles (range)	No. of aspects covered	Covered regression types	Covered multi-variable models	References
**1**	Revista Española de Cardiologia	Contemporary methods in biostatistics	2011	6	2	7.0 (7)	35	Linear, logistic and Cox	Yes	[22, 23]
**2**	Circulation	Statistical primer for cardiovascular research	2006–2009	23	3	6.0 (6)	31	Linear, logistic and Cox	Yes	[24–26]
**3**	Archives of Disease in Childhood	Statistics from the inside	1991–1995	16	3	4.7 (4–5)	30	Linear, and logistic	Yes	[27–29]
**4**	Deutsches Ärzteblatt International	Series of evaluation of scientific publications	2009–2017	24	1	7.0 (7)	27	Linear, logistic, Cox, and Poisson	Yes	[20]
**4**	European Journal of Cardio-Thoracic Surgery	Statistical primer	2018	5	1	6.0 (6)	27	Linear, logistic and Cox	Yes	[21]
**6**	American Journal of Roentgenology	Fundamentals of clinical research for radiologists	2000–2005	22	3	10.3 (4–16)	25	Linear, logistic and Cox	Yes	[15, 16, 30]
**6**	European Heart Journal	Statistical tutorials	2011–2014	5	1	8.0 (8)	25	Logistic and Cox	Yes	[31]
**8**	Nephrology Dialysis Transplantation	Clinical research in kidney diseases	2017	9	2	8.5 (8–9)	23	Linear, logistic, Cox, and Poisson	Yes	[32, 33]
**8**	Nature Methods	Points of significance	2013–2019	42	9	2.0 (2)	23	Linear and logistic	Yes	[34–42]
**8**	Journal of Thoracic Disease	Statistics corner	2015–2018	33	5	4.8 (2–6)	23	Linear, logistic and Cox	Yes	[43–47]
**11**	Critical Care	Statistics review		14	3	7.3 (6–9)	23		Yes	[48–50]
**12**	Radiology	Statistical concepts series	2002–2004	17	2	6.0 (6)	22	Linear and logistic	Yes	[51, 52]
**13**	Journal of Clinical Psychopharmacology	Statistics commentary series	2014–2019	32	2	3.0 (3)	19	Linear	Yes	[53, 54]
**14**	Kidney International	abc of epidemiology	2007–2009	17	3	5.0 (5)	15	Linear, logistic and Cox	Yes	[55–57]
**14**	Advances in Physiology Education	Explorations in statistics	2010–2018	13	1	6.0 (6)	15	Linear	No	[58]
**14**	Journal of the American Medical Association	JAMA guide to statistics and methods	2014–2017	20	2	2.0 (2)	15	Logistic	Yes	[59, 60]
**17**	The Medical Journal of Australia	Accessible series on statistics for clinicians	2016–2018	10	1	3.0 (3)	13	Linear and logistic	Yes	[61]
**17**	Nephron Clinical Practice	Kidney disease and population health	2009–2011	17	1	6.0 (6)	13	Cox	Yes	[62]
**17**	American Journal of Ophthalmology	Series on statistics	2008–2010	14	3	2.0 (2)	13	Linear, logistic, Cox and Poisson	Yes	[63–65]
**20**	British Medical Journal	Statistics notes	1994–2018	68	4	1.0 (1)	12	Linear	Yes	[66–69]
**21**	Nephrology Dialysis Transplantation	Clinical epidemiology in nephrology	2010–2013	9	2	5.5 (5–6)	11	Logistic	Yes	[70, 71]
**22**	Annals of Thoracic Surgery	The Statistician’s page	2001–2016	24	1	1.0 (1)	8	Logistic	Yes	[72]
**23**	Nutrition	Random bytes	1996–1997	8	2	1.5 (1–2)	3	Linear and logistic	No	[73, 74]

The most informative series with respect to our pre-specified list of aspects was published in *Revista Española de Cardiologia*, which mentioned 35 aspects in two articles on regression modeling (Table 1). Similarly, *Circulation* and *Archives of Disease in Childhood* covered 31 and 30 aspects in three article each. The number of articles and the years of publication varied across the series (Fig 2). Some series comprised only five articles whereas *Statistics Notes* of the *BMJ* published 68 short articles, which was very successful with some articles that were cited about 2000 times. Almost all series covered multivariable regression in at least one article. The range of regression types varied across series. Most statistical series were published with the intention to improve the knowledge of their readership about how to apply appropriate methodology in data analyses and how to critically appraise published research [17–19].

### Characteristics of articles

The top three articles that covered the highest number of aspects (27 to 34 out of 44 aspects) on six to seven pages were published in *Revista Española de Cardiologia*, *Deutsches Ärzteblatt International*, and in *European Journal of Cardio-Thoracic Surgery* [20–22]. The article of Nuñez et al. [22] published in *Revista Española de Cardiologia* covered the most popular regression types (linear, logistic and Cox regression) and explained not only general aspect but also gave insights into non-linear modeling and variable selection. Schneider et al. [20] covered all regression types that we considered in our review in their publication in *Deutsches Ärzteblatt International*. The top-ranked article in *European Journal of Cardio-Thoracic Surgery* [21] particularly focused on the development and validation of prediction models.

### Explanation of aspects in the series

**Fig 3:**
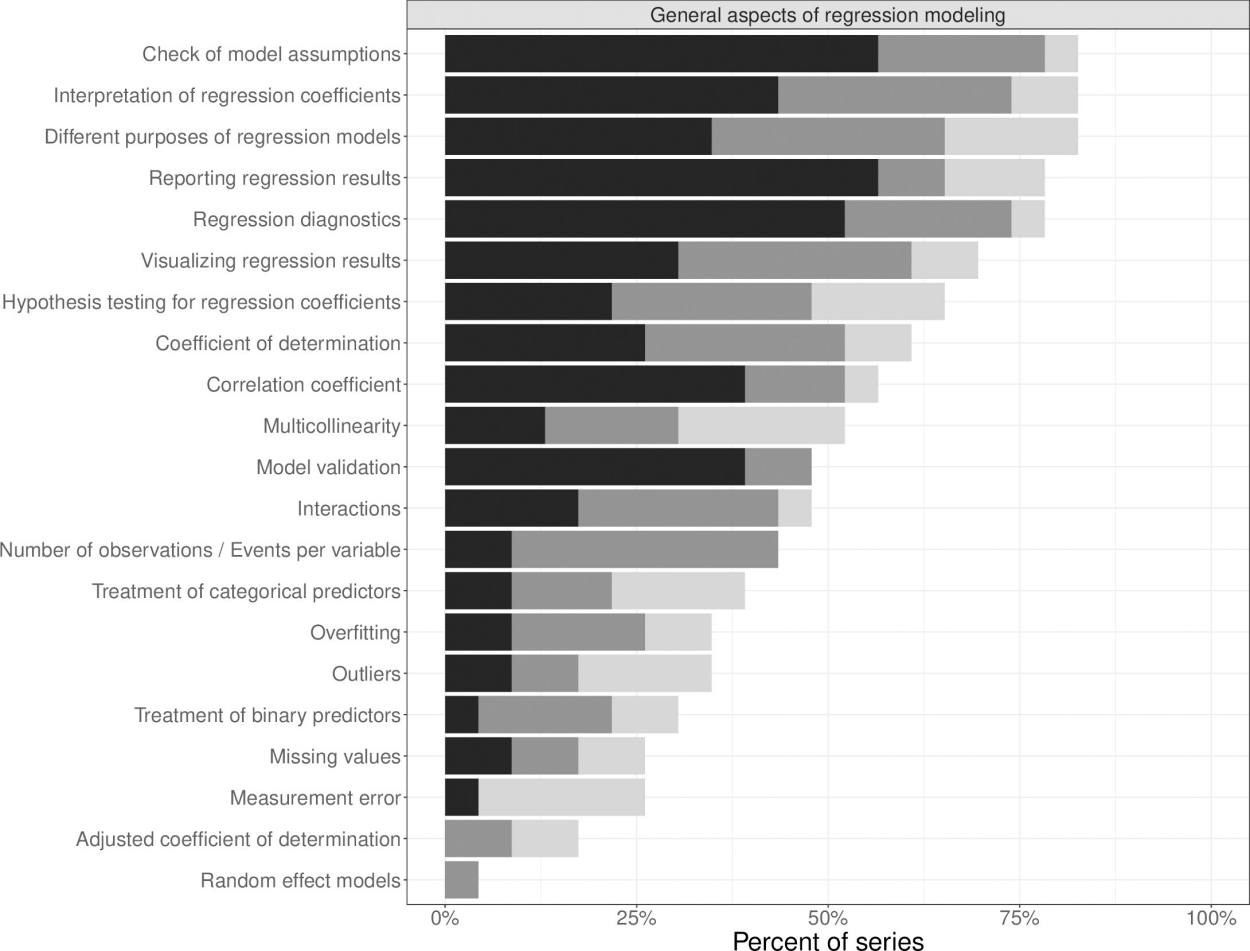
Extent of explanation of general aspects of regression modeling in statistical series: One sentence only (light grey), more than one sentence to one paragraph (grey) and more than one paragraph (black).

Almost all statistical series included at least one article that mentioned or explained *multivariable regression* (Table 1). *Logistic regression* was the most frequently described regression type in 19 out of 23 series (83%), followed by *linear regression* (78%). *Cox regression/survival model* (including proportional hazards regression) was mentioned in twelve series (52%) and was less extensively described than linear and logistic regression. *Poisson regression* was covered by three series (13%). Each of the considered general aspects of regression modeling were mentioned in at least four series (17%) (Fig 3) except for *random effect models*, which were treated in only one series (4%). *Interpretation of regression coefficients*, *model assumptions*, and *different purposes of regression mode* were covered in 19 series (83%). The aspect *different purposes of regression models* comprised at least one statement in an article concerning purposes of regression models, which could be identified by keywords like prediction, description, explanation, etiology, or confounding. More than one sentence was used for the explanation of different purposes in 15 series (65%). In 18 series (78%), *reporting of regression results* and *regression diagnostics* were described, which was done extensively in most series. Aspects like *treatment of binary covariates*, *missing values*, *measurement error*, and *adjusted coefficient of determination* were rather infrequently mentioned and found in four to seven series each (25–30%).

**Fig 4:**
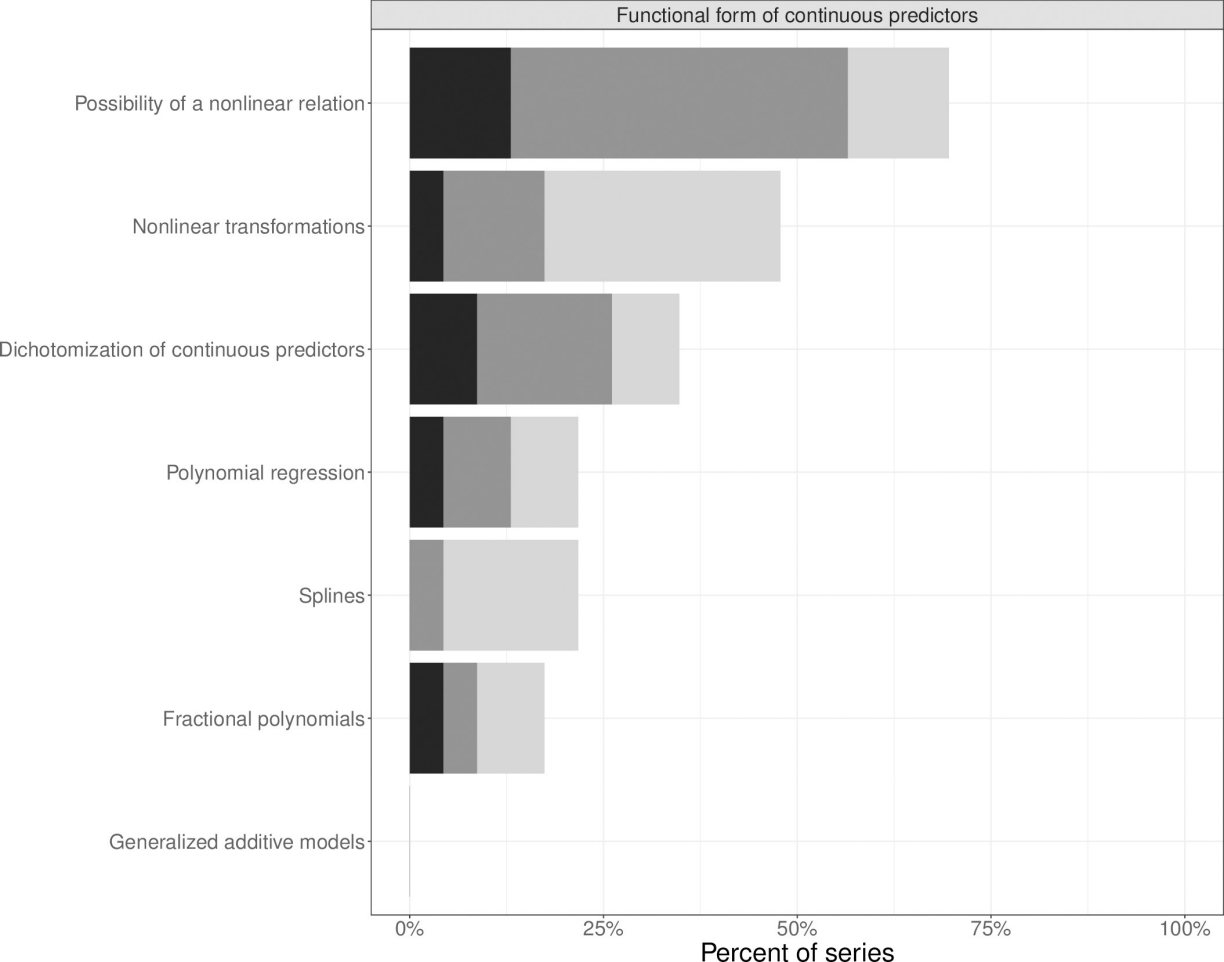
Extent of explanation of aspects of functional forms of continuous predictors in statistical series: One sentence only (light grey), more than one sentence to one paragraph (grey) and more than one paragraph (black).

At least one aspect of *functional forms of continuous predictors*, was mentioned in 17 series (74%), but details were hardly ever given (Fig 4). *The possibility of non-linear relation* and *non-linear transformations* were raised in 16 (70%) and eleven series (48%), respectively. *Dichotomization of continuous covariates* was found in eight series (35%) and it was extensively discussed in two (9%). More advanced techniques like the use of *splines* or *fractional polynomials* were mentioned in some series but detailed information for splines was not provided. *Generalized additive models* were never mentioned.

**Fig 5:**
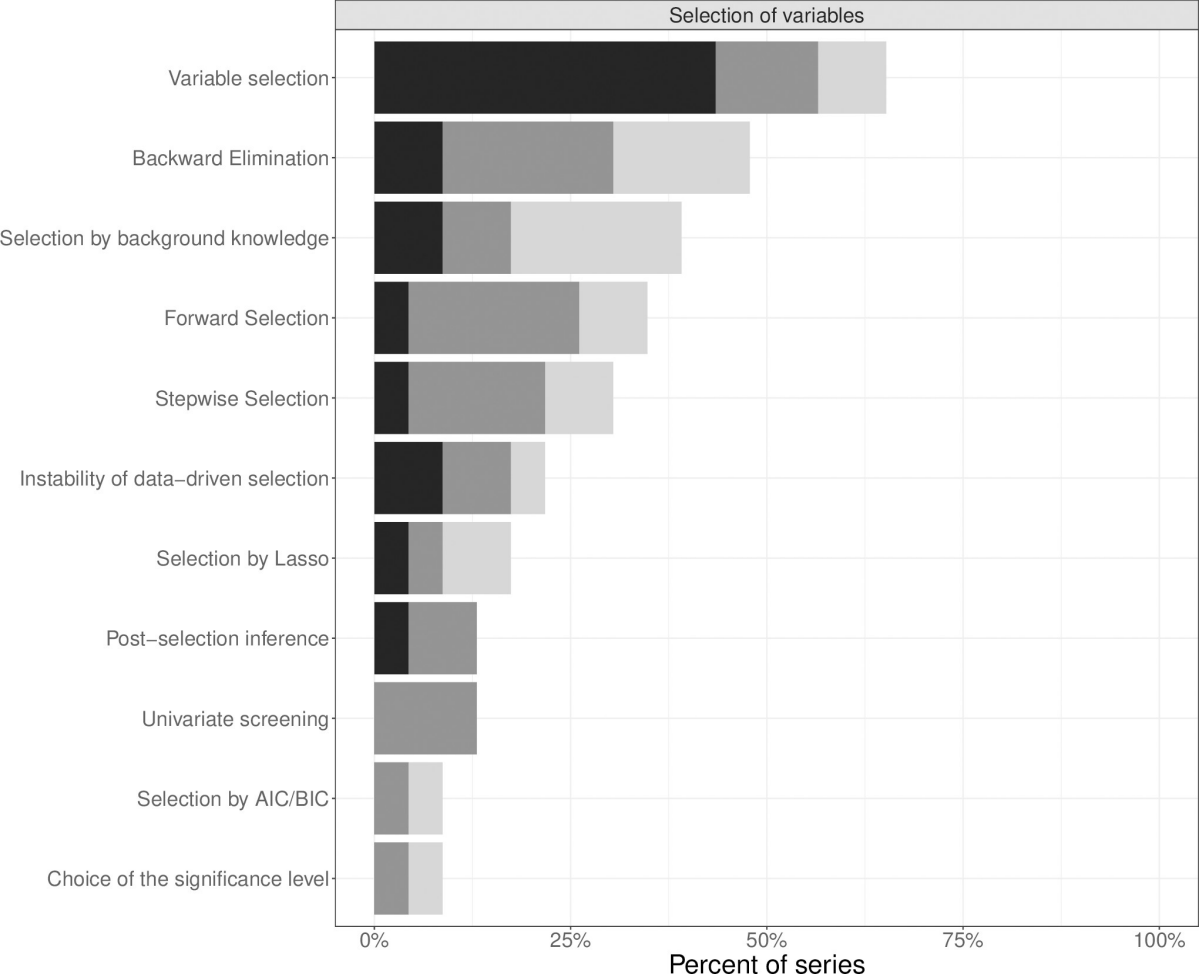
Extent of explanation of aspects of selection of variables in statistical series: One sentence only (light grey), more than one sentence to one paragraph (grey) and more than one paragraph (black).

*Selection of variables* was mentioned in 15 series (65%) and described extensively in ten series (43%) (Fig 5). However, specific variable selection methods were rarely described in detail. *Backward elimination*, *selection based on background knowledge*, *forward selection*, and *stepwise selection* were the most frequently described selection methods in seven to eleven series (30–48%). *Univariate screening*, which is still popular in medical research, was only described in three series (13%) in up to one paragraph. Other aspects of variable selection were hardly ever mentioned. *Selection based on AIC/BIC*, relating to best subset selection or stepwise selection based on these information criteria, and the *choice of the significance level* were found in 2 series only (9%). Relative frequencies of aspects mentioned in articles are detailed in Figs 1–3 in S5 File.

### Software

We found general recommendations for software in nine articles of nine different series. Authors mentioned R, Nanostat, GLIM package, SAS and SPSS [75–78]. SAS as well as R were recommended in three articles. In only one article the authors referred to a specific package in R. Detailed code examples were provided in two articles only [16, 58]. In the article of Curran-Everett [58], the R script file was provided as appendix and in the article of Obuchowski [16], code chunks were included throughout the text directly showing how to derive the reported results. In all, software recommendations were rare and mostly not detailed.

### Recommendations and warnings in the series

Recommendations and warnings were given on many aspects of our list. All statements are listed in S5 File: Table 1 and some frequent statements across articles are summarized below.

### Statements on general aspects

We found numerous recommendations and warnings on general aspects as described in the following. Concerning data preparation, some authors recommended to impute missing values in multivariable models, e.g. by multiple imputation [20–22, 31]. Steyerberg et al. [31] and Grant et al. [21] discouraged from using a complete case analysis to handle missing values. As an aspect of model development, *number of observations/events per variable* was a disputed topic in several articles [79–81]. In seven articles, we found explicit recommendations for the number of observations (in linear models) or the events per variable (in logistic and Cox/survival models), varying between at least ten to 20 observations/events per variable [16, 20, 22, 25, 31, 33, 55]. Several recommendations and warnings were given on *model assumptions* and *model diagnostics*. Many series authors recommended to check assumptions graphically [24, 27, 44, 58, 72] and they warned that models may be inappropriate if the assumptions are not met [20, 24, 31, 33, 52, 55, 56, 62]. In the context of Cox proportional hazards model, authors especially mentioned the proportional hazards assumption [24, 44, 49, 56, 62]. Concerning reporting of results, some authors warned to not confuse odds ratios with relative risks or hazard ratios [25, 44, 59]. Several warnings could also be found on reporting performance of a model. Most authors did not recommend to report the coefficient of determination R^2^ [20, 27, 51, 61] and indicated that the pitfall of R^2^ is that its value increases with increasing number of covariates in the model [15]. Schneider et al. [20] and Richardson et al. [61] recommended to use the adjusted coefficient of determination instead. We also found many recommendations and statements about *model validation* for prediction models. Authors of the evaluated articles recommended cross-validation or bootstrap validation instead of split sample validation if internal validation is performed [21, 22, 31, 70, 72]. It was also suggested that internal validation is not sufficient for the model to be used in clinical practice and an external validation should be executed as well [21]. In several articles, we found that authors warned about applying the Hosmer-Lemeshow test because of potential pitfalls [31, 60, 61]. For *reporting regression results*, in two articles the guideline for Transparent Reporting of multivariable prediction models for Individual Prognosis or Diagnosis (TRIPOD) was mentioned [21, 71, 82].

### Statements on functional form of continuous predictors

*Dichotomization of continuous predictors* is an aspect of *functional forms of continuous predictors* that was frequently discussed. Many authors argued against categorization of continuous variables because it may lead to loss of power, to increased risk of false positive results, to underestimation of variation, and to concealment of non-linearities [21, 26, 31, 69]. However, other authors advised to categorize continuous variables if the relation to the outcome is non-linear [24, 25, 59].

### Statements on variable selection

We also found recommendations in favor of or against specific variable selection methods. Four articles explicitly recommended to take advantage of background knowledge to select variables [15, 20, 48, 59]. Univariate screening was advised against by one article [19]. Comparing stepwise selection methods, Grant et al. [21] preferred backward elimination over forward selection. Authors warned about consequences of stepwise methods such as unstable selection and overfitting [21, 31]. It was also pointed out that selected models must be interpreted with greatest caution and implications should be checked on new data [28, 53].

### Methodological gaps in the series

This descriptive analysis of contents gives rise to some observations on important gaps and possibly misleading recommendations. First, we found that one general type of regression models, Poisson regression, was not treated in most series. This omission is probably due to the fact that Poisson regression is less frequently applied in medical research because most outcomes are binary or time-to-event and, therefore, logistic and Cox regression are more frequent. Second, several series introduced the possibility of non-linear relations of continuous covariates with the outcome. However, only few statements on how to deal with non-linearities by specifying flexible functional forms in multiple regression were available. Third, we did not find very detailed information on advantages and disadvantages of data-driven variable selection methods in any of the series. Finally, tutorials on statistical software and on specific code examples were hardly found in the reviewed series.

### Misleading recommendations in the series

Quality assessment of recommendations would have been controversial and we did not intend doing it. Nevertheless, here we mention two issues that we consider as severely misleading. Although univariate screening as a method for variable selection was never recommended in any of the series, one article showed an example with the application of this procedure to pre-filter the explanatory variables based on their associations with the outcome variable [47]. It is known since long that univariate screening should be avoided because it has the potential to wrongly reject important variables [83]. In another article it was suggested that a model can be considered robust if results from both backward elimination and forward selection agree [20]. Such agreement does not support robustness of stepwise methods: relying on agreement is a poor strategy [84, 85].

### Series and articles recommended to read

Depending on the aim of the planned study, as well as the focus and knowledge level of the reader, different series and articles might be recommended. The series in *Circulation* comprised three papers about multiple linear and logistic regression [24–26], which provide basics and describe many essential aspects of univariable and multivariable regression modeling. For more advanced researchers, we recommend the article of *Nuñ*ez et al. in *Revista Española de Cardiologia* [22], which gives a quick overview of aspects and existing methods including functional forms and variable selection. The *Nature Methods* series published short articles focusing on few, specific aspects of regression modeling [34–42]. This series might be of interest if one likes to spent more time on learning about regression modeling. If someone is especially interested in prediction models, we recommend a concise publication in the *European Heart Journal* [31], which provides details on model development and validation for predictive purposes. For the same topic we can also recommend the paper by Grant et al. [21]. We consider all series and articles recommended in this paragraph as suitable reading for medical researchers but this does not imply that we agree to all explanations, statements and aspects discussed.

## Discussion

### Summary and consequences for future work

This review summarizes the knowledge about regression modeling that is transferred through statistical tutorials published in medical journals. A total of 23 series with 57 topic-relevant articles were identified and evaluated for coverage of 44 aspects of regression modeling. We found that almost all aspects of regression modeling were at least mentioned in any of the series. Several aspects of regression modeling, in particular most general aspects, were covered. However, detailed descriptions and explanations of non-linear relations and variable selection in multivariable models were lacking. Only few papers provided suitable methods and software guidance for analysts with a relatively weak statistical background and limited practical experience as recommended by the STRATOS initiative [1]. However, we confess that currently there is no agreement on state of the art methodology [3].

Nevertheless, readers of statistical tutorials should not only be informed about the possibility of non-linear relations of continuous predictors with the outcome but they should also be given a brief overview about which methods are generally available and may be suitable. This could be achieved by tutorials that introduce readers to methods like fractional polynomials or splines, explaining similarities and differences between these approaches, e.g., by comparative, commented analyses of realistic data sets. Such documents could also show how alternative analyses (considering/ignoring potential non-linearities) may result in conflicting results and explain the reasons for such discrepancies.

Detailed tutorials on variable selection could aim at describing the mechanism of different variable selection methods, which can easily be applied with standard statistical software, and should state in what situations variable selection methods are needed and could be used. For example, if sufficient background knowledge is available, prefiltering or even the selection of variables should be based on this information rather than using data-driven methods on the entire data set. Such tutorials should provide comparisons and interpretation of the results of various variable selection methods and suggest adequate methods for different data settings.

Generally, the articles also lacked details on software to perform statistical analysis and usually did not provide code chunks, descriptions of specific functions, an appendix with commented code or references to software packages. Future work should also focus on filling this gap by recommendations of software as well as providing well commented and documented code for different statistical methods in a format that is accessible by non-experts. We recommend that software, packages and functions therein to apply certain methods should be reported in every statistical tutorial article. The respective code to derive analysis results could be provided in an appendix or directly in the manuscript text, if not too lengthy. Any provided code in the appendix should be well-structured and lavishly commented referring to the particular method and describing all defined parameter settings. This will encourage medical researchers to increase the reproducibility of their research by also publishing their statistical code, e.g., in electronic appendices to their publications. For example, worked examples with openly accessible data sets and commented code allowing fully reproducible results have a high potential to guide researchers in their own statistical tasks. On the contrary, we discourage from using point-and-click software programs, which sometimes output far more analysis results than requested. Users may pick inadequate methods or report wrong results inadvertently, which could debilitate their research work.

Generally, our review may stimulate the development of targeted gap-filling guidance and tutorial papers in the field of regression modeling, which should support medical researchers in several ways: 1) by explaining how to interpret published results correctly, 2) by guiding them how to critically appraise the methodology used in a published article, 3) by enabling them to plan, perform basic statistical analyses and report results in a proper way and 4) by helping them to identify situations in which the advice of a statistical expert is required. In S3 File: CRF article screening we commented which aspects should usually be addressed by an expert and which aspects are considered basic.

### Strengths and limitations

According to our knowledge this is the first review on series of statistical tutorials in the medical field with the focus on regression modeling. Our review followed a pre-specified and published protocol to which many experienced researchers in the field of applied regression modeling contributed. One aspect of this contribution was the collection of series of statistical tutorials that could not be identified by common keyword searches.

We standardized the selection process by designing an inclusion checklist for series of statistical tutorials and by providing a manual for the content form with which we extracted the actual information of the article and series. Another strength is that the data collection process was performed objectively since each article was analyzed by two out of three independent raters. Discrepancies were discussed among all three of them to find a consent. This procedure avoided that single opinions were transferred to the output of this review. This review is informative for many clinical colleagues who are interested in statistical issues in regression modeling and search for suitable literature.

This review also has limitations. An automated, systematic search was not possible because series could not be identified by common keywords neither on the series’ title level nor on the article’s title level. Thus, not all available series may have been found. To enrich our initial query, we also searched on certain journals’ webpages and requested our expert panel from the STRATOS initiative to complement our list with other series they were aware of. We also included series that were suggested by one reviewer during the peer-review procedure of this manuscript. This selection strategy may impose a bias towards higher-quality journals since series of less prestigious journals might not be known to the experts. However, the higher-quality journals can be considered as the primary source of information for researchers seeking advice on statistical methodology.

We considered only series with at least five articles. This boundary is of course to a certain extend arbitrary. It was motivated by the fact that we intended to do analyses on the series level, which is only reasonable if a series covers an adequate number of articles. We also assumed that larger series are more visible and well-known to researchers.

We also might have missed or excluded some important aspects of regression modeling in our catalogue. The catalogue of aspects was developed and discussed by several experienced researchers of the STRATOS initiative working in the field of regression modeling. After submission of the protocol paper some more aspects were added on request of its reviewers [7]. However, further important aspects such as meta-regression, diagnostic models, causal inference, reproducibility or open data and open software code were not addressed. We encourage researchers to repeat similar reviews on these related fields.

A third limitation is that we only searched for series whereas there might be other educational papers on regression modeling that were published as single articles. However, we believe that the average visibility of an entire series and thereby its educational impact is much higher than for isolated articles. This does not negate that there could be excellent isolated articles, which can have a high impact for training medical researchers. While working on the final version of this paper we became aware of the series *Big-data Clinical Trial Column* in the *Annals of Translational Medicine*. Until 1 January 2019 they had published 36 papers and the series would have been eligible for our review. Obviously, we might have overseen further series, but it is unlikely that it has a larger effect on the results of our review.

Moreover, there are many introductory textbooks, educational workshops and online video tutorials, some of them with excellent quality, which were not considered here. A detailed review of such sources clearly was out of our scope.

## Conclusion

Despite many series of statistical tutorials being available to guide medical researchers on various aspects of regression modeling, several methodological gaps still persist, specifically on addressing nonlinear effects, model specification and variable selection. Furthermore, papers are published in a large number of different journals and are therefore likely unknown to many medical researchers. This review fills the latter gap, but many more steps are needed to improve the quality and interpretation of medical research. More detailed statistical guidance and tutorials with a low technical level on regression modeling and other topics are needed to better support medical researchers who perform or interpret regression analyses.

## Supporting information

S1 Checklist PRISMA reporting guideline. (PDF)

S1 File List of candidate series for potential inclusion in the review. (PDF)

S2 File Case report form–series inclusion. (PDF)

S3 File Case report form–article screening. (PDF)

S4 File Manual for the article screening sheet. (PDF)

S5 File Supplementary figures and tables. (PDF)

S1 Data Collected data. (XLSX)
