# Supplementary material for: Review of guidance papers on regression modeling in statistical series of medical journals
Source: PLoS One. 2022 Jan 24;17(1):e0262918. doi: 10.1371/journal.pone.0262918 (PMC8786189; doi:10.1371/journal.pone.0262918)
Supplement: S1 File — (PDF) [file pone.0262918.s002.pdf]

List of candidate series for potential inclusion in the review

Selected series are shaded grey.

ID	Journal	Title of the series	Reason for exclusion
1	Advances in Physiology Education	Explorations in statistics column	
2	American Journal of Epidemiology	Practice of Epidemiology Issue Section	too advanced
3	Annals of Allergy, Asthma & Immunology	The reading, writing, and arithmetic of the medical literature	less than 5 coherent articles
4	Archives of Disease in Childhood	Statistics from the inside series	
5	Circulation	Statistical Primer for Cardiovascular Research	
6	Clinical Radiology	A guide to clinical epidemiology for radiologists: Part I and II	less than 5 coherent articles
7	Columbia University	Population Health Methods	not published in a medical journal
8	Deutsche Medizinische Wochenzeitschrift	Statistik-Serie in der DMW	not published in English
9	Deutsche Zahnärztliche Zeitschrift	EbM-Splitter	not published in English
10	Deutsches Ärzteblatt	Serie zur Bewertung wissenschaftlicher Publikationen	not published in English
11	Deutsches Ärzteblatt International*	Series on Evaluation of Scientific Publications	
12	Emergency Medicine Journal	An introduction to statistics	no topic-relevant article
13	European Heart Journal	Statistical Tutorials	
14	JAMA	Guide to Statistics and Methods	
15	JAMA	Users' Guide to the Medical Literature	no topic-relevant article
16	Journal of Clinical Oncology	Statistics in Oncology Series	too advanced
17	Journal of Medical Systems	Methodological and Statistical Techniques: What Do Residents Really Need to Know About Statistics?	less than 5 coherent articles

18	Journal of the American College of Surgeons	Interpreting statistics in medical literature: A vade mecum for surgeons	less than 5 coherent articles
19	Journal of Thoracic Disease	Statistics Corner Column	
20	Kidney International	ABC of Epidemiology	
21	Malawi Medical Journal	Statistics Corner	less than 5 coherent articles
22	Medizinische Monatszeitschrift für Pharmazeuten	Fortbildung Wissensbasierung	not published in English
23	Nederlands Tijdschrift van Geneeskunde		not published in English
24	Nephrology Dialysis Transplantation	Clinical research in Kidney Diseases	
25	Nephrology Dialysis Transplantation	Clinical Epidemiology in Nephrology	
26	Nephron Clinical Practice	Kidney Disease and population health	
27	Postgraduate Medical Journal	Development of the Biostatistics and Clinical Epidemiology Skills	less than 5 coherent articles
28	RadioGraphics	Statistics 101 for radiologists	less than 5 coherent articles
29	Radiology	Statistical Concepts Series	
30	Statistics in Medicine	Tutorials in Biostatistics Series	too advanced
31	The Annals of Thoracic Surgery	The statistician's page	
32	The BMJ	Statistics Notes	
33	The European Journal of Cardio-Thoracic Surgery	Statistics Primers	
34	The Medical Journal of Australia	Accessible series on statistics for clinicians	
35	American Journal of Roentgenology	Fundamentals of Clinical Research for Radiologist	
36	American Journal of Ophthalmology	Series on Statistics	
37	Annals of Internal Medicine	Research and Reporting Methods	too advanced
38	Circulation	Primer on Statistical Interpretation or Methods	less than 5 coherent articles
39	Critical Care	Statistical review	
40	International Journal of Epidemiology	Education Corner	too advanced

41	Journal of Clinical Psychopharmacology	Statistics Commentary Series	
42	Nature Methods	Points of Significance	
43	Nutrition	Random Bytes	
44	Psychosomatic Medicine	Statistical Corner	less than 5 coherent articles
45	Revista Española de Cardiología	Contemporary Methods in Biostatistics	
46	Seminars in Hematology	Interpretation of Quantitative Research	less than 5 coherent articles
47	The Journal of Thoracic and Cardiovascular Surgery	Statistics for the Rest of Us	no topic-relevant article

*Articles of *Deutsches Ärzteblatt International* were originally written in German and published in *Deutsches Ärzteblatt*. These articles are later translated to English for *Deutsches Ärzteblatt International*.
