# Supplementary material for: Review of guidance papers on regression modeling in statistical series of medical journals
Source: PLoS One. 2022 Jan 24;17(1):e0262918. doi: 10.1371/journal.pone.0262918 (PMC8786189; doi:10.1371/journal.pone.0262918)
Supplement: S2 File — (PDF) [file pone.0262918.s003.pdf]

Regression Modeling in Medical Statistics
Review of Statistical Series

Case Report Form - Series inclusion

**Name of rater**	
**Date of rating**	

**Journal**	
**Journal impact factor (Scopus, 2018)**	
**Statistical series name (if applicable)**	
**Overall number of articles (until 1.1.2019)**	

**Inclusion criteria (series)**	Y	N
1. Published in a medical journal	☐	☐
2. Statistical series with 5 or more coherent articles	☐	☐
3. Written in English	☐	☐
4. Target audience with limited background in statistics	☐	☐
5. The series contains at least one topic-relevant article	☐	☐
**Are all inclusion criteria met?**	☐	☐

We consider an article as topic-relevant, if:

a) Its title contains one of the key words “regression”, “linear”, “logistic“, “Cox”, “survival”, “Poisson”, “multivariable” or “multivariate” or
b) It is fairly plausible from the title that the article deals with regression modeling even if the title does not contain one of the above keywords.

Identified topic-relevant articles *(sort according to year of publication, start with oldest article)*

**No**	**Rank**	**Title**	**First author**	**Year of publication**
1			*First name, Last name*	
2				
3				
4				
5				
6				
7				
8				

Comments

E.g. None or Does not qualify because it is a single article and not a series.
