# Supplementary material for: Review of guidance papers on regression modeling in statistical series of medical journals
Source: PLoS One. 2022 Jan 24;17(1):e0262918. doi: 10.1371/journal.pone.0262918 (PMC8786189; doi:10.1371/journal.pone.0262918)
Supplement: S3 File — (PDF) [file pone.0262918.s004.pdf]

Regression Modeling in Medical Statistics
Review of Statistical Series

Case Report Form - Article Screening

**Name of rater**	
**Date of rating**	

**Journal**	
**Statistical series**	
**Title of the article**	
**Rank of the article within the series**	
**Author(s) of the article**	
**Year of publication of the article**	

**Aspect number**		Explained (y/n)	Extent of explanation (S/M/L)	Example provided (y/n)	Software advice given (y/n)	Recommendation given[1] (y/n)	Warning issued[1] (y/n)	Rater comment[2] (y/n)
**1**	**Type of regression model**							
1.1	Univariable regression							
1.2	Multivariable regression							
1.3	Linear regression							
1.4	Logistic regression							
1.5	Cox regression / survival model							
1.6	Poisson regression							
1.7								
1.8								
**2**	**General aspects of regression modeling**							
2.1	Different purposes of regression models							
2.2	Interpretation of regression coefficients							
2.3	Check of model assumptions							
2.4	Correlation coefficient							
2.5	Coefficient of determination							
2.6	Adjusted coefficient of determination							
2.7	Treatment of binary predictors							
2.8	Treatment of categorical predictors							
2.9	Hypothesis testing for regression coefficients							
2.10	Multicollinearity							
2.11	Interactions							
2.12	Outliers							
2.13	Missing values							
2.14	Measurement error							
2.15	Overfitting							
2.16	Number of observations / Events per variable							

[1] Please write down any recommendation or warning in the comment section at the end of the sheet.
[2] Please indicate if you have a comment on the explanation of a particular aspect.

Regression Modeling in Medical Statistics
Review of Statistical Series

Case Report Form - Article Screening

Aspect number		Explained (y/n)	Extent of explanation (S/M/L)	Example provided (y/n)	Software advice given (y/n)	Recommendation given (y/n)	Warning issued (y/n)	Rater comment (y/n)
2.17	Visualizing regression results							
2.18	Random effect models							
2.19	Regression diagnostics							
2.20	Model validation							
2.21	Reporting regression results							
2.22								
2.23								
**3**	**Functional form of continuous predictors**							
3.1	Possibility of a nonlinear relation							
3.2	Dichotomization of continuous predictors							
3.3	Nonlinear transformations							
3.4	Polynomial regression							
3.5	Fractional polynomials							
3.6	Splines							
3.7	Generalized additive models							
3.8								
3.9								
**4**	**Selection of variables**							
4.1	Selection by background knowledge							
4.2	Univariate screening							
4.3	Forward selection							
4.4	Backward Elimination							
4.5	Stepwise selection							
4.6	Choice of the „significance level“							
4.7	Selection by AIC / BIC							
4.8	Selection by Lasso							
4.9	Instability of data-driven selection							
4.10	Post-selection inference							
4.11								
4.12								

Comments

Refers to: Please specify Aspect number/ general comment

Basic aspects are highlighted in blue and advanced aspects are highlighted in orange. Medical researchers should be aware of the existence of those advanced aspects. However, if such aspects are encountered or are needed to be addressed in studies, statistical experts should be consulted and may conduct the statistical analysis.
