# Supplementary material for: Review of guidance papers on regression modeling in statistical series of medical journals
Source: PLoS One. 2022 Jan 24;17(1):e0262918. doi: 10.1371/journal.pone.0262918 (PMC8786189; doi:10.1371/journal.pone.0262918)
Supplement: S4 File — (PDF) [file pone.0262918.s005.pdf]

Regression Modeling in Medical Statistics
Review of Statistical Series

Manual for the article screening sheet

Explained (yes/no)

Please indicate whether a particular aspect is explained (mentioned or covered at all).

Extent of explanation (S/M/L)

Please judge the extent of the explanation. We define:

- S:=Short:=Up to one sentence
- M:=Medium:=More than one sentence and up to about one paragraph
- L:=Long:=More than one paragraph

Example provided (yes/no)

Please indicate whether a particular aspect is illustrated by an example.

Software advice given (yes/no/not applicable)

Please indicate whether software advice is provided for a particular aspect enabling a practitioner to conduct the analysis on her/his own.

Recommendation given (yes/no/not applicable)

Please indicate whether there is a recommendation for a particular aspect.

Warning issued (yes/no/not applicable)

Please indicate whether there is a warning issued against a particular aspect.

Rater comment (y/n)

Please indicate whether you have a comment concerning a particular aspect. Please write down:

- Recommendations given
- Warnings issued
- Incorrect explanations
- Any other things that you find remarkable

in the comment section at the end of the sheet. To indicate that the comment refers to a particular aspect, use the corresponding identifier at the beginning of the comment (e.g. 1.3 or 2.2). Please use quotation marks ("…"), if you directly quote from the article.

Further aspects

You can add further aspects/key words of regression modeling not listed so far.

Comments section

Besides comments on particular aspects, you can also specify comments of a more general nature. Please use the key word "general comment" in this case. If you need more space for your comments, you can simply expand the comments section (in Word) or write on the backside of the sheet (if you have printed the sheet).
