# Supplementary material for: Review of guidance papers on regression modeling in statistical series of medical journals
Source: PLoS One. 2022 Jan 24;17(1):e0262918. doi: 10.1371/journal.pone.0262918 (PMC8786189; doi:10.1371/journal.pone.0262918)
Supplement: S5 File — (PDF) [file pone.0262918.s006.pdf]

S5 File

Review of guidance papers on regression modeling in statistical series of medical journals

Christine Wallisch, Paul Bach, Lorena Hafermann, Nadja Klein, Willi Sauerbrei, Ewout W. Steyerberg, Georg Heinze, Geraldine Rauch on behalf of topic group 2 of the STRATOS initiative

Content

Supplementary Figure 1 2

Supplementary Figure 2 3

Supplementary Figure 3 4

Supplementary Table 1 5

References 36

Supplementary Figure 1: Extent of explanation of *general aspects of statistical modeling* in articles: up to one sentence (lightgrey), up to one paragraph (grey) and more than one paragraph (black).

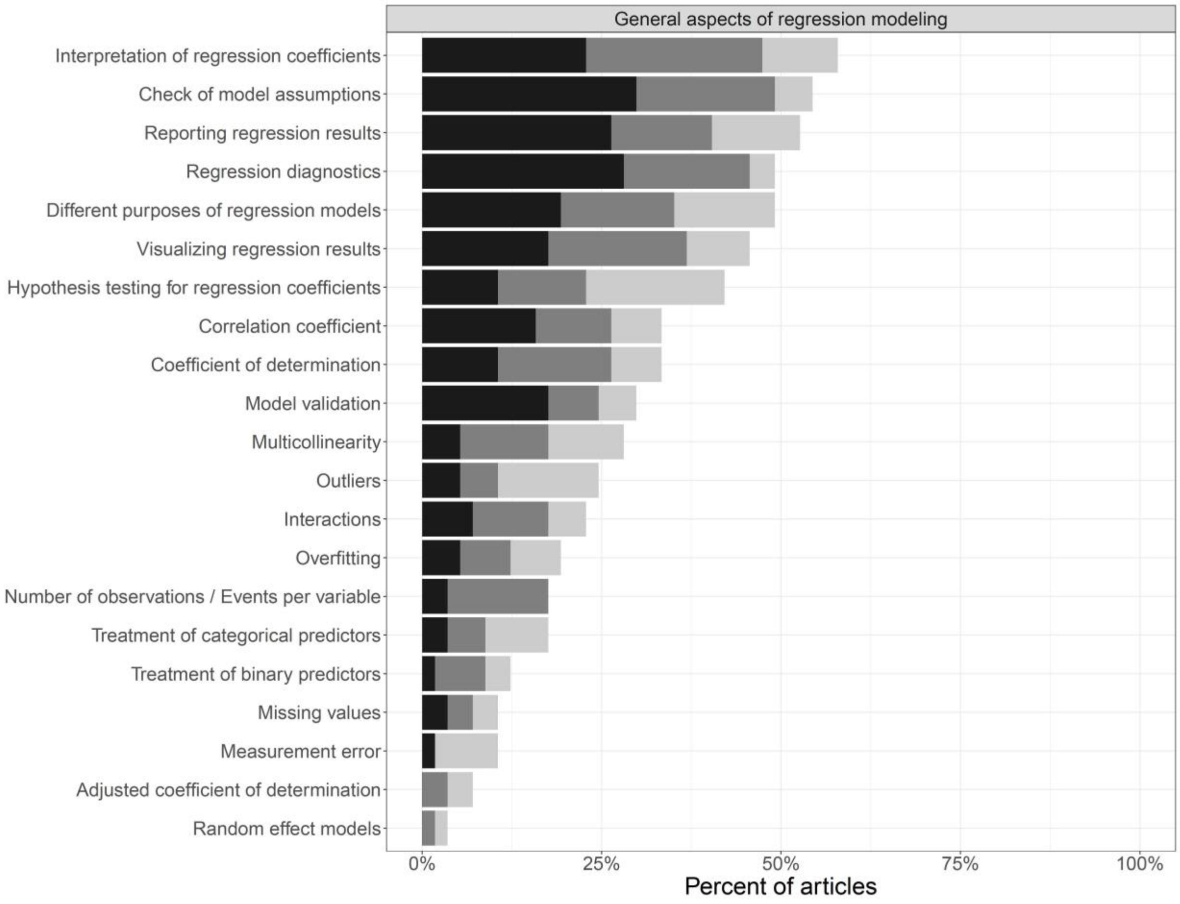

Supplementary Figure 2: Extent of explanation of aspects of *functional forms of continuous predictors* in articles: up to one sentence (lightgrey), up to one paragraph (grey) and more than one paragraph (black).

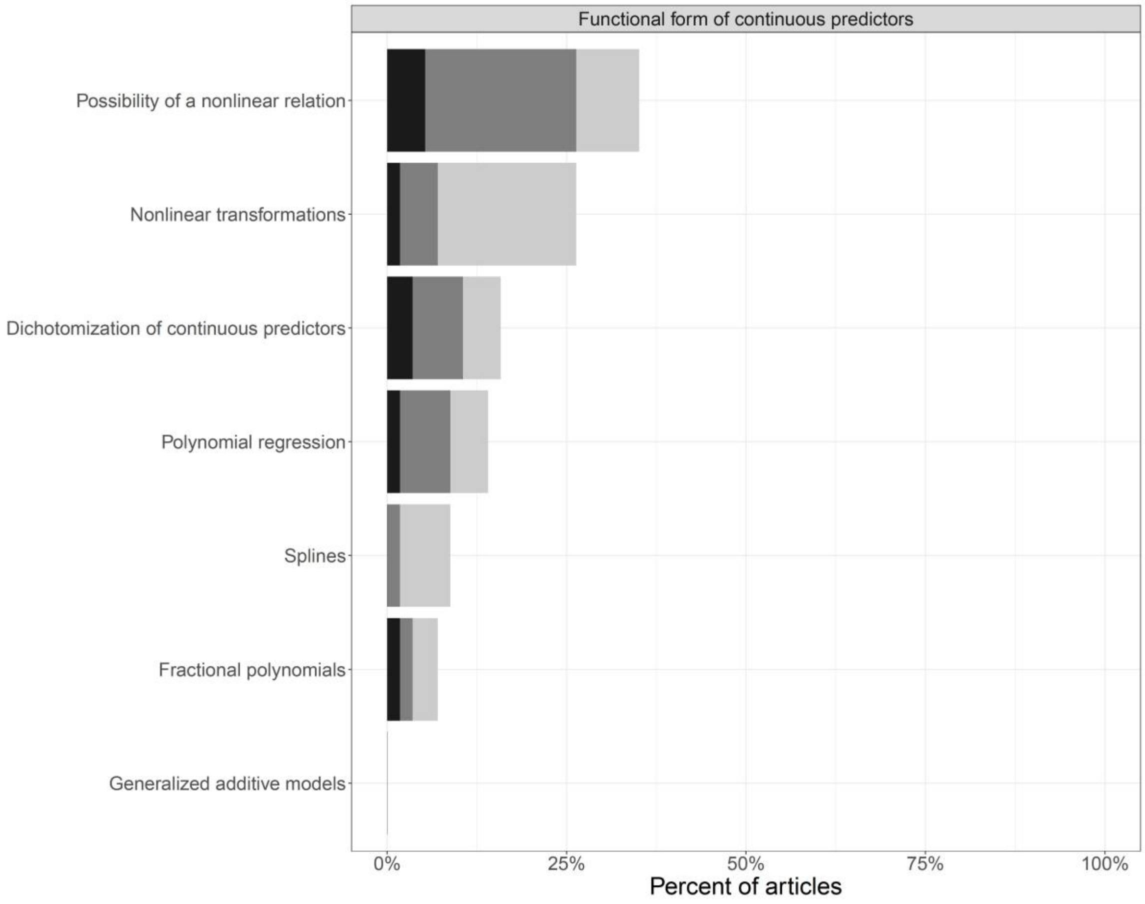

Supplementary Figure 3: Extent of explanation of aspects of *selection of variables* in articles: up to one sentence (lightgrey), up to one paragraph (grey) and more than one paragraph (black).

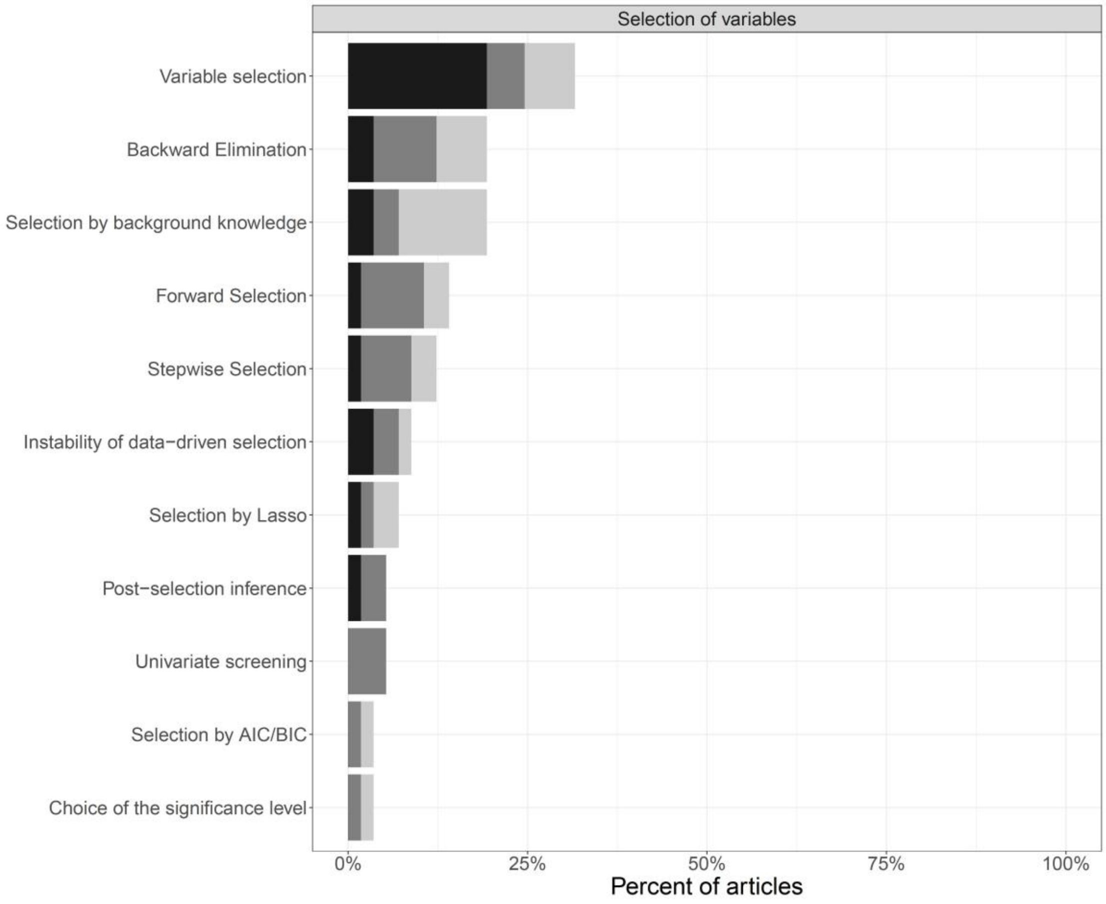

Supplementary Table 1: Recommendations and warnings reported in the articles.

No.	Aspect	Recommendation	Warning
**1**	**Type of regression model**		
1.1	Univariable regression		
1.2	Multivariable regression	„We want to reach correct conclusions not only about which predictors are important and the size of their effects but also about the structure by which multiple predictors simultaneously relate to the response. […] A series of simple regressions cannot accomplish these tasks." Use multivariable regression instead of many univariable regression models to reach correct conclusions." [1]<br>"Linear regression modeling is not used as frequently in medical research as logistic regression, as clinicians often prefer to dichotomize continuous outcomes. It can still be quite informative, though, to run linear regression on the continuous outcome as supplementary analysis." [2]	„No matter how strong a relationship is demonstrated with regression analysis, it should not be interpreted as causation." [3]<br>"The regression should not be used to predict or estimate outside the range of values of the independent variable of the sample." [3]<br>"However, even including several inputs into the model the 'exact' response value can never be established." [4]

1.3	Linear regression		"Even when an estimated regression line provides a good fit to the observed data, it is important not to extrapolate beyond the range of the sample, because the estimated line may not be appropriate." [5]
1.4	Logistic regression	"As the OR is a symmetric effect measure (Table 2), logistic regression is the model of choice in case control designs where subjects are selected retrospectively based on disease status." [6]	"The associations found through logistic regression models are intended to provide insights into what might happen in a similar population of future patients. Certain combinations of patient characteristics and factors may have been sparsely represented in the data set (eg, young patients with sepsis and a low Glasgow Coma Scale score but a normal blood pressure and respiratory rate), and the estimates of the model for mortality among such patients should be considered with caution." [7]<br>"A second limitation of logistic regression is that the variables must have a constant magnitude of association across the range of values for that variable." [7]<br>"Therefore, logistic regression should be considered as an alternative to Cox regression only when the duration of the cohort follow-up can be disregarded for being too short, or

			when the proportion of censoring is minimal and similar between the two levels of the explanatory variable.” [8]
1.5	Cox regression	“While popular and the default method of many software programs, the Breslow approximation has shown to be less accurate than Efron method in many situations. The Efron approximation is generally the recommended method.“ [9]<br>“For risk estimation in prospective longitudinal studies Poisson and Cox’s regressions are the methods of choice.” [6]<br>“In settings such as the current example, where the goal is to estimate the effect of treatment adjusting for othercovariates, it often is useful to provide a plot of the model-based covariate-adjusted survival function for the 2 treatment groups.“ [10]	“Censored observations are those who survived at least as long as they remained in the study but for whom their actual event-free survival times are *not known exactly*. Such right-censored survival times underestimate the true (but unknown) time to event.“ [6]
1.6	Poisson regression		

**2**	**General aspects of regression modeling**	

2.1	Different purposes of regression models	“Although modelling strategies help identify multiple relationships, their direction and temporal sequence should be made explicit in the design and ideally tested in experimental studies.” [4]	“As explained in the above exposition, prediction results should never be interpreted causally.” [11]<br>“The results of the analysis, however, need to be interpreted with care, particularly when looking for a causal relationship or when using the regression equation for prediction.” [12]<br>“The interpretation of logistic regression shares some similarities with that of linear regression; for instance, variables given the greatest importance may be reliable predictors but might not actually be causal.” [13]
2.2	Interpretation of regression coefficients		“The odds ratio is sometimes confused with the relative risk, which is the ratio of probabilities rather than odds.” [14]<br>“The proper interpretation of the regression coefficient thus requires attention to the units of measurement.” [15]<br>“Because probabilities are more intuitive than ORs, it is important to avoid confusing them.” [7]<br>“However, HR, RR and OR are estimates of different nature and should not be confused.” [9]

2.3 Check of model assumptions	“Residual plots help us decide if our provisional statistical model is appropriate; they are essential to a thorough regression analysis.” [16]<br>“This is best done graphically.” [17]<br>“This additivity assumption can be relaxed by including statistical interaction terms.” [18]<br>„If a covariate violates the proportional hazards assumption, several solutions can be applied:<br>• Stratify on this covariate: then there won’t be any estimation of HR for this variable;<br>• Add an interaction between the covariate and time.” [9]<br>“…if the survival curves of two groups cross, the HR is clearly not the same over time, and in that case the use of the Cox regression model with proportional hazards is inappropriate. “ [19]<br>“The log rank test and Cox’s proportional hazards model assume that the hazard ratio is constant over time. Care must be taken to check this assumption.” [20]	“For example, we may want to investigate the relation between two variables and take several pairs of readings from each of a group of subjects. Such data violate the assumption of independence inherent in many analyses, such as t tests and regression. Researchers sometimes put all the data together, as if they were one sample. Most statistics textbooks do not warn the researcher not to do this.” [21]<br>“If these assumptions are incorrect, the model may be invalid, and the interpretation of the data that is based on that model may be incorrect.” [22]<br>“Obviously, critical violations of model assumptions would make the model inappropriate.“ [4]

2.4	Correlation coefficient	"r should be reported together with a P value" [23]	"A formula exists for the standard error of a sample correlation, but this is not useful for two reasons-the formula involves the unknown correlation, and in addition the distribution of the sample coefficient is liable to be far from Normal." [24]<br>"Estimates of correlation and R^2 depend not only on the magnitude of the underlying true association but also on the variability of the data included in the sample." [1]<br>"Correlation analysis is generally overused. It is often interpreted incorrectly (to establish "causation") and should be reserved for generating hypotheses rather than for testing them." [3]<br>"High correlation may indicate a strong association but not causation." [25]<br>"The observed correlation (or lack of it) may be due to a confounding variable." [25]<br>"Correlation between aggregate values is stronger than at the individual level." [25]

		“Correlation is influenced by the range of the X and Y variables” [25]<br>“High correlation does not mean measurement equivalence.” [25]<br>“Association should not be confused with causality”. [23]
2.5	Coefficient of determination	“The multiple correlation coefficient is a leftover from the early days of statistics, when correlation and coefficients for measuring it were all rage, and it is nowadays best avoided.” [17]<br>“The coefficient of determination can easily be made artificially high by including a large number of independent variables in the model. The more independent variables one includes, the higher the coefficient of determination becomes. This, however, lowers the precision of the estimate (estimation of the regression coefficients bi).” [15]<br>“However, because is there no direct equivalent to R2 in logistic regression, many variations of pseudo-R2 have been developed by different statisticians, each with a slightly different interpretation.” [26]

			“The R^2 value is a broadly useful measure of how good the model is; however, it has a couple of pitfalls. Its validity depends on model assumptions being correct, and its value increases as the number of explanatory variables increases, even if these are not related to the outcome.” [27]<br>“In interpreting these results, it must be noted that the R^2 statistic is influenced by the number of predictor variables in the model” [25]
2.6	Adjusted coefficient of determination	“Instead of the raw (uncorrected) coefficient of determination, the corrected coefficient of determination should be given.” [15]<br>“The R^2value can be adjusted to combat this increase” [27]	
2.7	Treatment of binary predictors		
2.8	Treatment of categorical predictors		“… it would be totally concealed by an analysis which treated the social class codes as if they were values of a continuous measurement.” [26, 28]

2.9	Hypothesis testing for regression coefficients		
2.10	Multicollinearity	"Predictors that are highly correlated are unlikely to contribute significant independent information to the multivariable model and one or the other should generally be excluded." [29]	"This problem is called multicollinearity and should be of concern if the correlation between a pair of predictor variables is above about 0.9" [1]<br>"If you see any Variance Inflation Factor (VIF) greater than 10 (although some people use 5), you have a problem." [30]<br>„If VIF(X*i*) is large, then there may be high variation in the regression coefficient estimate between different samples—for example, when VIF > 10, the regression coefficients should not be interpreted." [31]
2.11	Interactions	"[…] limit the number of interactions, and include only those prespecified and based on biological plausibility" [8]	"Although significant interaction terms may be identified, inclusion of them in the model does not necessarily improve model performance." [29]<br>"When this is not true and the value of one predictor alters the effect of another, there is said to be an "interaction" between the 2 predictors. Such interactions need to be

			explicitly included in the analysis to ensure the estimated associations are valid.” [7]<br>“Hence, in the presence of interactions, the main effects cannot be interpreted by themselves.” [22]
2.12	Outliers	“However, they may have arisen purely by chance and be a result of biological variability.In this case, removing them would lead to underestimation of the variability in the data and unduly influence inference.” [32]	“…influential observations can lead to erroneous results, and therefore their presence and effect should be evaluated and understood.” [1]
2.13	Missing values	“Whenever the value of either a dependent or an independent variable is missing, this particular observation has to be excluded from the regression analysis. […] There are a number of ways to deal with the problem of missing values.” incl. reference [15]<br>“We recognize that imputation should be performed carefully, but is usually preferable to a complete case analysis.” [18]<br>“Multiple imputation, which maintains the size of the data set available for model development, is the preferred	“By default, patients with any missing value are excluded from statistical analyses (complete case analysis or available case analysis). This is inefficient since available information of other predictors is lost.” [18]<br>“A complete case analysis can substantially reduce the data available for model development and lead to inaccurate estimates of specific predictors or overall model performance.” [29]

		approach but relies on the assumption that the data are missing at random." [29]<br>"impute data if necessary as sample size is important" [8]
2.14	Measurement error	
2.15	Overfitting	
2.16	Number of observations / Events per variable	"A rule of thumb for stability of the estimates from logistic regression is to have at least 10 events (or nonevents, whichever is rarer in the data) per predictor in the model – more precisely, per degree of freedom used in the model)" [14]<br>"In general, the number of observations should be at least 20 times greater than the number of variables under study." [15]<br>"… a common rule of thumb is to require at least 10 events per variable (EPV)." [18]<br>"A critical question is how many covariates can be entered into a multiple linear regression analysis. The number of

covariates allowed depends on the sample size. A practical rule is to include 1 covariate every 10 observations." [33]

"A simple rule is to include in the multiple logistic regression model 1 covariate every 10 events." [33]

"Most authors recommend that there should be at least 10 to 20 times as many observations as there are coefficients in the model; otherwise the estimates are very unstable. Models of binary outcomes require at least 10 events per parameter." [4]

"A general rule of thumb with logistic regression analysis is that you need at least 10–15 observations (here, patients) of each type (here, type is patients with a particular lesion pathology) for each predictor variable in the model."[34]

"As with any statistical modeling, we must be careful not to overfit the model (i.e., include more predictor variables than can be supported by the number of observations in the study)."[34]

""Large sample sizes are required for logistic regression to provide sufficient numbers in both categories of the response variable. The more explanatory variables, the larger the sample size required. With small sample sizes, the Hosmer–Lemeshow test has low power and is unlikely

		todetect subtle deviations from the logistic model. Hosmer and Lemeshow recommend sample sizes greater than 400."[35]<br>"In linear multiple regression, a minimum of 10 to 15 observations per predictor has been recommended. For survival models, the number of events is the limiting factor (10 to 15). For logistic regression, if the number of non-events is smaller than the number of events, then it will become the number to be used. In simulation studies, 10 to 15 events per variable were the optimal ratio." [8]
2.17	Visualizing regression results	"In my statistics course, I announce that there are four rules for any statistical analysis: 1. Plot the data. 2. Study the data. 3. Analyze the data. 4. Analyze the analysis." [16]<br>"The initial judgment of a possible relationship between two continuous variables should always be made on the basis of a scatter plot (scatter graph)." [15]<br>"When analyzing survival data, the survival curves should always be plotted using the KM method (and not using the Cox regression method)." [19]<br>"In settings such as the current example, where the goal is to estimate the effect of treatment adjusting for

		othercovariates, it often is useful to provide a plot of the model-based covariate-adjusted survival function for the 2 treatment groups.“ [12]	
2.18	Random effect models	“This mixed-model regression approach is usually necessary to correctly estimate uncertainty when repeated observations exist within subjects” [1]<br>“The standard form of logistic regression presented here also presumes that observations are independent. This would not be the case for longitudinal or clustered data, and analyzing such data as independent could give misleading conclusions. Methods such as generalized estimating equations or random-effects models can be used for such data.“ [14]	
2.19	Regression diagnostics	“Residual plots help us decide if our provisional statistical model is appropriate; they are essential to a thorough regression analysis.”[16]<br>“Nevertheless the graphical analysis of the logistic regression model is a tool that all analysts should consider using when the logistic regression is crucial to the analysis of a clinical data series.” [36]	“The odds ratio values given above describe the model as it is applied to the data. If the model and the data are not in good agreement, then these odds ratios are not very meaningful.” [14]<br>"Performing a linear regression makes sense only if the relationship is linear.“ [15]

“A more searching examination of the goodness of fit of the regression involves inspection of the individual residuals, which we have seen in table 2 (any statistical package worthy of the name will calculate these for you). This is best done graphically.“ [17]

"In simple linear regression, one can assess linearity by looking at a plot of the data points. In multiple regression, one can examine scatterplots of Y and of the residuals versus the individual predictor variables." [1]

"Multiple regression assumes that the residuals are normally distributed and have equal variance across the predictor data space. These assumptions are typically evaluated with the use of graphical methods and related statistics to assess the residuals." [1]

"Identify outliers and influential observations whose influence on the estimates and goodness of fit should be analyzed.” [37]

“If the two survival curves remain parallel and don’t intersect, we can assume in a first approach the proportional hazard.” [9]

“The relationship between continuous variables and survival is assumed to be linear. If continuous predictors are included in the model, this assumption must be checked.” [9]

"The validity of any conclusion drawn by using these methods is critically dependent on the ascertainment of a series of assumptions. The lack of a rigorous validation of these conditions may lead to flawed data analyses and invalid results.“ [33]

"Although in practice it is unlikely that the proportional hazards assumption is ever fully satisfied, important violation of the PH assumption may result in wrong and misleading estimates.” [38]

"Although in practice it is unlikely that the proportional hazards assumption is ever fully satisfied, an important violation of the proportional hazards assumption may result in wrong and misleading estimates.” [19]

	“Plotting the residuals is a method for graphically detecting non-linearity (residuals are computed from the observed values minus estimated values).” [9]<br>“Often crossing survival curves are a strong indication of nonproportionality.“ [38]<br>“For example, if the survival curves of two groups cross, the HR is clearly not the same over time, and in that case the use of the Cox regression model with proportional hazards is inappropriate. Two popular approaches to test if the hazards are proportional are described elsewhere.“ [19]<br>“To understand whether the assumptions have been met, determine the magnitude of the gap between the data and the assumptions of the model.“ [3]	
2.20 Model validation	“Bootstrapping also cannot replace validation by a new study. In spite of these limitations, bootstrapping is a useful and easily implemented technique that should be considered by all analysts.”[36]	“Using a random sample for model development, and the remaining patients for validation (‘split sample validation’) is a common, but suboptimal form of internal validation.” [18]<br>“Considering such groups with their deviations from the ideal line makes the plot a graphical illustration of the often used

“Rather, we emphasize the older recalibration idea as proposed by Cox in 1958. Perfect predictions should be on the ideal line, described with an intercept alpha (‘A’) of 0 and slope beta(‘B’) of 1. The log odds of predictions are used as the predictor of the 0/1 outcome, or the log (hazard) for time-to-event outcomes.” [18]

“It is therefore advised to consider a range of thresholds when quantifying the clinical usefulness of a prediction model.” [18]

““Better methods are cross-validation and bootstrap resampling,” [18]

“Risk prediction models should be both internally and externally validated before they are adopted in clinical practice.” [29]

“The preferred approach for internal validation is to use bootstrapping or k-fold cross-validation.” [29]

“If a model demonstrates poor discrimination on external validation, then it is likely that a new model is required; however, if a model demonstrates poor calibration, it can Hosmer–Lemeshow goodness-of-fit test. We do not recommend this test for assessment of calibration. It does not indicate the direction of any miscalibration and only provides a P-value for differences between observed and predicted endpoints per group of patients (commonly deciles).Such grouping is arbitrary and imprecise, and P-values depend on the combination of the extent of miscalibration and sample size.”[18]

“The calibration slope B is often smaller than 1 if a model was developed in a relatively small data set. Such a finding reflects that predictions were too extreme: low prediction too low, and high predictions too high.” [18]

“Calibration and discrimination are important aspects of a prediction model, and consider the full range of predicted risks. However, these aspects do not assess clinical usefulness, i.e. the ability to make better decisions with a model than without. […] It is usually difficult to define a threshold since empirical evidence for the relative weight of benefits and harms is often lacking.” [18]

potentially be updated or recalibrated. If a model consistently demonstrates poor calibration, then it is likely that a new model is required.” [29]

“Face validity and clinical usefulness should be considered alongside statistical performance for all risk prediction models designed to be applied in clinical practice.” [29]

“External validation, applying the nomogram to an independent sample, is preferred to examine model generalizability. Alternatively, most studies tend to evaluate nomograms by internal validation, of which the bootstrapping method is one of the most reliable solutions.” [39]

“[…] validate the final model for calibration and discrimination, preferably using bootstrapping, and i) use shrink age methods if validation shows over-optimistic predictions.” [8]

“In sum, we recommend the ‘‘a, b, c’’ rule for the evaluation of predictions, with a (the intercept) and b

“If a model does not accurately discriminate, then it is not useful as a risk prediction model. Calibration is an assessment of how closely.” [29]

“The Hosmer–Lemeshow test is often used to assess model calibration and involves splitting the cohort, often into 10 equally sized groups, with contributing X^2 statistics from each group then summed to give an overall P-value. However, the test is influenced by the sample size, the number of groups and provides no information on the direction or magnitude of miscalibration.” [29]

“It is important that the same model building steps used to develop the model are replayed in the bootstrapping or cross-validation. […] An alternative internal validation approach, whereby the data are randomly split into development and validation data, is inefficient. For small to moderately sized data, it reduces the sample size for model development, therefore increasing the chances of overfitting, and leaves too few data to evaluate the model.“ [29]

(slope) referring to calibration, and c to the AUC (Fig. 2)." [40]

"The associations found through logistic regression models are intended to provide insights into what might happen in a similar population of future patients. Certain combinations of patient characteristics and factors may have been sparsely represented in the data set (eg, young patients with sepsis and a low Glasgow Coma Scale score but a normal blood pressure and respiratory rate), and the estimates of the model for mortality among such patients should be considered with caution.“ [7]

"If a model demonstrates poor discrimination on external validation, then it is likely that a new model is required; however, if a model demonstrates poor calibration, it can potentially be updated or recalibrated. If a model consistently demonstrates poor calibration, then it is likely that a new model is required." [29]

"If the definitions of the predictors or outcomes are unclear or ambiguous, then this will raise concerns about the face validity and limit the application of the model.“ [29]

“One of many formal tests is the Hosmer-Lemeshow test, where a high P value indicates a better fit. [...]. A poor fit may indicate the exclusion of important explanatory variables. However, this test is dependent on user-selected groups and, depending on your data, other tests may be more appropriate.” [27]

“A particular model might discriminate well, correctly identifying patients who are at higher risk than others, but fail to accurately estimate the absolute probability of an outcome.” [26]

“In addition, the Hosmer-Lemeshow statistic depends on the number of risk groups into which the study population is divided. There is no theoretical basis for the “correct” number of risk groups into which a population should be divided. Also, with sample sizes smaller than 500, the test has low power and can fail to identify poorly calibrated models.“ [26]

“It is important to remember that each predictive model, including the nomogram, is mathematically optimized to best-fit the data on which it was originally built. Hence, whether a

			nomogram can be used in practice will depend on whether it has good generalizability with other samples.” [39]<br>“A naive internal validation, computing performance measures in the same cohort that has been used to develop the model, usually leads to over-optimistic estimates of the performance of a prediction model.“ [41]<br>„If the number of individuals in the cohort is relatively low, or to avoid spurious results caused by one particular random split, more computer-intensive techniques based on many repeated splits of the data, like bootstrap or 10-fold cross-validation, should be applied for assessing the prognostic performance of the same risk prediction model.“ [41]
2.21	Reporting regression results	“As a final step we propose to consider is the presentation of a prediction model, such that it best addresses the clinical needs.” [18]<br>“When developing a risk model, it is important that the full prediction model with all regression coefficients and the model intercept is published” [29]	“It is sometimes tempting to not report the nonsignificant end points and report only the statistically significant ones. This strategy, however, can lead to serious misinterpretations of the data because the type 1 error rate is not properly controlled.” [34]

“As a result, the Transparent Reporting of a multivariable prediction model for Individual Prognosis or Diagnosis (TRIPOD) recommendations were developed and published in 2015. The TRIPOD guidelines are a checklist of 22 items deemed essential for transparent reporting of a prediction model study and are designed to improve the quality of risk prediction model research.” [29]

“Reported ORs for the effects of predictors should be accompanied by 95 % confidence intervals” [7]

“Of importance, the discrimination and the calibration should be reported with confidence intervals.” [42]

“The recent Strengthening the Reporting of Observational Studies in Epidemiology (STROBE) Statement advices a transparent presentation of the separate effect of each exposure as well as the joint effect, each relative to the unexposed group as (joint) reference.” [43]

“Such observations have led to the development of the Transparent reporting of a multivariable prediction model for individual prognosis or diagnosis (TRIPOD) guideline for

		reporting prediction studies, which has been adopted by many leading medical journals. Adherence to this guideline allows journals and readers to adequately assess the quality and usefulness of a prediction study, thereby reducing research waste.“ [11]<br>“As a main result of Cox regression analysis, one should present both the unadjusted and adjusted HRs with the corresponding 95% CIs.“ [19]
**3**	**Functional form of continuous predictors**	
3.1	Possibility of a nonlinear relation	“In a simple linear regression, one can assess linearity by looking at a plot of the data points. In multiple regression, one can examine scatterplots of Y and of residuals versus the individual predictor variables.” [1]<br>“The initial judgment of a possible relationship between two continuous variables should always be made on the basis of a scatter plot (scatter graph).This type of plot will show whether the relationship islinear (Figure 1) or nonlinear (Figure 2).” [15]

3.2	Dichotomization of continuous predictors	“Instead of categorizing continuous variables, we prefer to keep them continuous.” [44]<br>“When a variable is continuous, treating it as a continuous variable typically retains more information than collapsing it to an ordinal categorical variable. In some cases, however, the latter version maybe preferable” [5]<br>“For example, if the logarithm of the odds against the predictor X has a U shape […] splitting the predictor values into categories and using dummy variables to code for the categories may improve the fit” [14]<br>“If the association is not consistent over the age range, then age may be stratified into ranges (eg, 21-50, 51-65, and _66) based on the assumption that within each category, the influence of age will be similar.” [7]	“Dichotomising leads to several problems. Firstly, much information is lost, so the statistical power to detect a relation between the variable and patient outcome is reduced. Indeed, dichotomising a variable at the median reduces power by the same amount as would discarding a third of the data. Deliberately discarding data is surely inadvisable when research studies already tend to be too small. Dichotomisation may also increase the risk of a positive result being a false positive. Secondly, one may seriously underestimate the extent of variation in outcome between groups, such as the risk of some event, and considerable variability may be subsumed within each group. Individuals close to but on opposite sides of the cutpoint are characterised as being very different rather than very similar. Thirdly, using two groups conceals any non-linearity in the relation between the variable and outcome. Presumably, many who dichotomise are unaware of the implications.” [44]<br>“When a variable is continuous, treating it as a continuous variable typically retains more information than collapsing it

		to an ordinal categorical variable. In some cases, however, the latter version may be preferable." [5] "We emphasize that continuous predictors should not be dichotomized (categorization as below vs. above a certain cut-off) in the model development phase, since valuable information is lost." [18] "However dichotomization of a continuous variable should be avoided as this can reduce the power by approximately the same amount as discarding one-third of the data." [29]
3.3	Nonlinear transformations	"However, any data transformation changes the meaning of the model parameters and their interpretation may become obscure." [4]
3.4	Polynomial regression	"In principle this can be fitted as a multiple regression equation, with xl =t, x2=t2 and so on. In practice there are difficulties. When higher powers are introduced, the successive terms can become closely collinear, leading to large standard errors." [17]
3.5	Fractional polynomials	

3.6	Splines		
3.7	Generalized additive models		
**4**	**Selection of variables**	"Elastic net offers the best of both worlds and can be used to create a simpler model that will likely perform better on new data." [45]	"It is important, however, to avoid rote application of these methods, particularly for large data sets containing many possible predictor variables in which multicollinearity may be a problem." [1]
4.1	Selection by background knowledge	„Variable selection should be carried out on the basis of medical expert knowledge and a good understanding of biometrics." [15]<br>„Ideally, all biologically relevant factors should be included." [7]<br>"Several models may produce equally good statistical fits for a set of data and it is therefore important when choosing a model to take account of biological or clinical considerations and not depend solely on statistical results." [35]	

		"The choice of model should always depend on biological or clinical considerations in addition to statistical results."[35] "Although criteria such as the R2 and BIC may be used to assess modelfit, the choice of which predictor variables go into a model depends also on their clinical relevance, their impact on the magnitude of regression coefficients associated with the remaining predictors, and their statistical significance." [25]	
4.2	Univariate screening		„However, excluding potentially useful risk factors merely because they are not significantly associated with the outcome on univariable analysis is not recommended." [29]
4.3	Forward Selection	„The evaluation of a regression model requires the performance of both forward and backward selection of variables. If these two procedures result in the selection of the same set of variables, then the model can be considered robust." [15]	"Backward model selection where all predictors are included at first and predictors are subsequently removed is generally preferred to forward model selection, whereby the model is built up by adding predictors in starting with the strongest predictor. Although stepwise selection may be useful, a

			potential limitation of model selection strategies is that it can lead to overfitting of the model.” [29]
4.4	Backward Elimination	“The evaluation of a regression model requires the performance of both forward and backward selection of variables. If these two procedures result in the selection of the same set of variables, then the model can be considered robust.“ [15]<br>“Backward model selection where all predictors are included at first and predictors are subsequently removed is generally preferred to forward model selection…” [29]<br>“If used, proceed with a backward elimination instead, and set thecriterion for stopping rule equivalent to AIC (P = .157)” [8]	
4.5	Stepwise Selection		“Stepwise selection methods are widely used to reduce a set of candidate predictors, but have many disadvantages. In particular, when the numbers of events are low, the selection is instable, the estimated regression coefficients are too extreme, and the performance of the selected model is overestimated” [18]

			"First, there are many statistical tests computed in the background, in order to determine which variable to enter or remove at each stage. With even a small number of IVs, there can be scores or even hundreds of tests performed. What this means is that we have lost all control over the levels, in that as we increase the number that are calculated, the probability of chance significance (ie, a Type 1 error) increases exponentially." [30]<br>"It is for these reasons that Leigh states that "stepwise is unwise."" [30]<br>"„What the reader should look out for is the use of stepwise procedures (be very, very leery of the results), [...]" [30]
4.6	Choice of the „significance level"	"Relaxing the P = .05 value used as the stopping rule improves the selection of important variables in small datasets." [8]	"The stopping rules ('F to remove' and so on) are almost entirely arbitrary, and the ostensible significance levels are so untrustworthy as to be positively misleading." [26] – also concerns 4.10
4.7	Selection by AIC/BIC	"If used, proceed with a backward elimination instead, and set thecriterion for stopping rule equivalent to AIC (P = .157)" [8]	
4.8	Selection by Lasso		"The interpretation of logistic regression shares some similarities with that of linear regression; for instance,

		variables given the greatest importance may be reliable predictors but might not actually be causal."[45]<br>"Note that even with large values of λ, parameter magnitudes are reduced but not set to zero." [45]
4.9	Instability of data-driven selection	"But (and there's always a 'but') these advantages are more than offset by the problems created by stepwise procedures. First, there are many statistical tests computed in the background, in order to determine which variable to enter or remove at each stage. With even a small number of IVs, there can be scores or even hundreds of tests performed. What this means is that we have lost all control over the P levels, in that as we increase the number that are calculated, the probability of chance significance (ie, a Type 1 error) increases exponentially. Indeed, some simulations have concluded that up to 75% of the variables selected by stepwise techniques may in fact be noise or "garbage" variables, not at all related to the DV and which won't appear in the equation if the study is replicated.<br>The bigger problem is that stepwise procedures may mislead us when we try to interpret the final regression equation." [30]
4.10	Post-selection inference	"A regression equation with a small number of covariates selected from a larger set must be interpreted with the

		greatest caution. If at all possible, its implications should be checked using a separate sample of data from the one used in the calculations." [28]<br>"But (and there's always a 'but') these advantages are more than offset by the problems created by stepwise procedures. First, there are many statistical tests computed in the background, in order to determine which variable to enter or remove at each stage. With even a small number of IVs, there can be scores or even hundreds of tests performed. What this means is that we have lost all control over the P levels, in that as we increase the number that are calculated, the probability of chance significance (ie, a Type 1 error) increases exponentially. Indeed, some simulations have concluded that up to 75% of the variables selected by stepwise techniques may in fact be noise or "garbage" variables, not at all related to the DV and which won't appear in the equation if the study is replicated.<br>The bigger problem is that stepwise procedures may mislead us when we try to interpret the final regression equation." [30]
**General**	"The take-away lesson for those running a regression is to always collaborate with a statistician." [30]	

References

1. Slinker BK, Glantz SA. Multiple linear regression - Accounting for multiple simultaneous determinants of a continuous dependent variable. Circulation. 2008;117(13):1732-7. doi: 10.1161/Circulationaha.106.654376.
2. Boscardin WJ. The use and interpretation of linear regression analysis in ophthalmology research. Am J Ophthalmol. 2010;150(1):1-2. Epub 2010/07/09. doi: 10.1016/j.ajo.2010.02.022.
3. Zou KH, Tuncali K, Silverman SG. Correlation and simple linear regression. Radiology. 2003;227(3):617-22. doi: 10.1148/radiol.2273011499.
4. Ravani P, Parfrey P, Gadag V, Malberti F, Barrett B. Clinical research of kidney diseases III: Principles of regression and modelling. Nephrol Dial Transpl. 2007;22(12):3422-30. doi: 10.1093/ndt/gfm777.
5. Crawford SL. Correlation and regression. Circulation. 2006;114(19):2083-8. doi: 10.1161/Circulationaha.105.586495.
6. Ravani P, Parfrey P, Murphy S, Gadag V, Barrett B. Clinical research of kidney diseases IV: standard regression models. Nephrol Dial Transpl. 2008;23(2):475-82. doi: 10.1093/ndt/gfm880.
7. Tolles J, Meurer WJ. Logistic regression relating patient characteristics to outcomes. Jama-J Am Med Assoc. 2016;316(5):533-4. doi: 10.1001/jama.2016.7653.
8. Nuñez E, Steyerberg EW, Nuñez J. Regression modeling strategies. Rev Esp Cardiol. 2011;64(6):501-7. Epub 2011/05/03. doi: 10.1016/j.recesp.2011.01.019.
9. Brembilla A, Olland A, Puyraveau M, Massard G, Mauny F, Falcoz PE. Use of the Cox regression analysis in thoracic surgical research. J Thorac Dis. 2018;10(6):3891-6. doi: 10.21037/jtd.2018.06.15.
10. Hosmer DW, Jr., Lemeshow S. Survival analysis: applications to ophthalmic research. Am J Ophthalmol. 2009;147(6):957-8. Epub 2009/05/26. doi: 10.1016/j.ajo.2008.07.040.
11. van Diepen M, Ramspek CL, Jager KJ, Zoccali C, Dekker FW. Prediction versus aetiology: common pitfalls and how to avoid them. Nephrol Dial Transpl. 2017;32:1-5. doi: 10.1093/ndt/gfw459.
12. Bewick V, Cheek L, Ball J. Statistics review 7: Correlation and regression. Crit Care. 2003;7(6):451-9. doi: 10.1186/cc2401.
13. Lever J, Krzywinski M, Altman N. Logistic regression. Nature Methods. 2016;13(7):541-2. doi: 10.1038/nmeth.3904.
14. LaValley MP. Logistic regression. Circulation. 2008;117(18):2395-9. doi: 10.1161/Circulationaha.106.682658.
15. Schneider A, Hommel G, Blettner M. Linear regression analysis. Part 14 of a series on evaluation of scientific publications. Dtsch Arztebl Int. 2010;107(44):776-82. doi: 10.3238/arztebl.2010.0776.
16. Curran-Everett D. Explorations in statistics: regression. Adv Physiol Educ. 2011;35(4):347-52. doi: 10.1152/advan.00051.2011.
17. Healy MJR. 15. Multiple regression. Arch Dis Child. 1995;73(2):177-81. doi: 10.1136/adc.73.2.177.
18. Steyerberg EW, Vergouwe Y. Towards better clinical prediction models: seven steps for development and an ABCD for validation. Eur Heart J. 2014;35(29):1925-+. doi: 10.1093/eurheartj/ehu207.
19. Stel VS, Dekker FW, Tripepi G, Zoccali C, Jager KJ. Survival analysis II: Cox regression. Nephron Clin Pract. 2011;119(3):C255-C60. doi: 10.1159/000328916.
20. Bewick V, Cheek L, Ball J. Statistics review 12: Survival analysis. Crit Care. 2004;8(5):389-94. doi: 10.1186/cc2955.
21. Bland JM, Altman DG. Statistics notes 1. Correlation, regression, and repeated data. Brit Med J. 1994;308(6933):896. doi: 10.1136/bmj.308.6933.896.

22. Gareen IF, Gatsonis C. Primer on multiple regression models for diagnostic imaging research. Radiology. 2003;229(2):305-10. doi: 10.1148/radiol.2292030324.
23. Altman N, Krzywinski M. Association, correlation and causation. Nat Methods. 2015;12(10):899-900. Epub 2015/12/22. doi: 10.1038/nmeth.3587.
24. Healy MJR. 7. Regression and correlation. Arch Dis Child. 1992;67(10):1306-9. doi: 10.1136/adc.67.10.1306.
25. Dendukuri N, Reinhold C. Correlation and regression. AJR Am J Roentgenol. 2005;185(1):3-18. Epub 2005/06/24. doi: 10.2214/ajr.185.1.01850003.
26. Meurer WJ, Tolles J. Logistic regression diagnostics understanding how well a model predicts outcomes. Jama-J Am Med Assoc. 2017;317(10):1068-9. doi: 10.1001/jama.2016.20441.
27. Richardson AM, Joshy G, D'Este CA. Understanding statistical principles in linear and logistic regression. Med J Australia. 2018;208(8):332-+. doi: 10.5694/mja17.00222.
28. Healy MJR. 16. Multiple regression (2). Arch Dis Child. 1995;73(3):270-4. doi: 10.1136/adc.73.3.270.
29. Grant SW, Collins GS, Nashef SAM. Statistical Primer: developing and validating a risk prediction model. Eur J Cardio-Thorac. 2018;54(2):203-8. doi: 10.1093/ejcts/ezy180.
30. Streiner DL. Statistics Commentary Series: Commentary No. 32: Multiple Regression: What Can Possibly Go Wrong? J Clin Psychopharmacol. 2019;39(3):200-2. Epub 2019/03/29. doi: 10.1097/JCP.0000000000001040.
31. Altman N, Krzywinski M. Regression diagnostics. Nature Methods. 2016;13(5):385-6. doi: DOI 10.1038/nmeth.3854.
32. Altman N, Krzywinski M. Analyzing outliers: influential or nuisance? Nat Methods. 2016;13(4):281-2. Epub 2016/08/03. doi: 10.1038/nmeth.3812.
33. Tripepi G, Jager KJ, Dekker FW, Zoccali C. Linear and logistic regression analysis. Kidney Int. 2008;73(7):806-10. doi: 10.1038/sj.ki.5002787.
34. Obuchowski NA. Multivariate statistical methods. AJR Am J Roentgenol. 2005;185(2):299-309. Epub 2005/07/23. doi: 10.2214/ajr.185.2.01850299.
35. Bewick V, Cheek L, Ball J. Statistics review 14: Logistic regression. Crit Care. 2005;9(1):112-8. doi: 10.1186/cc3045.
36. Anderson WN. Statistical techniques for validating logistic regression models. Ann Thorac Surg. 2005;80(4):1169-. doi: 10.1016/j.athoracsur.2005.06.049.
37. Mengual-Macenlle N, Marcos PJ, Golpe R, Gonzalez-Rivas D. Multivariate analysis in thoracic research. J Thorac Dis. 2015;7(3):E2-E6. doi: 10.3978/j.issn.2072-1439.2015.01.43.
38. van Dijk PC, Jager KJ, Zwinderman AH, Zoccali C, Dekker FW. The analysis of survival data in nephrology: basic concepts and methods of Cox regression. Kidney Int. 2008;74(6):705-9. doi: 10.1038/ki.2008.294.
39. Liu RZ, Zhao ZR, Ng CSH. Statistical modelling for thoracic surgery using a nomogram based on logistic regression. J Thorac Dis. 2016;8(8):E731-E6. doi: 10.21037/jtd.2016.07.91.
40. Steyerberg EW, Van Calster B, Pencina MJ. Performance measures for prediction models and markers: evaluation of predictions and classifications. Rev Esp Cardiol. 2011;64(9):788-94. Epub 2011/07/19. doi: 10.1016/j.recesp.2011.04.017.
41. Tripepi G, Heinze G, Jager KJ, Stel VS, Dekker FW, Zoccali C. Risk prediction models. Nephrol Dial Transpl. 2013;28(8):1975-80. doi: 10.1093/ndt/gft095.
42. Riley RD, Ensor J, Snell KIE, Harrell FE, Martin GP, Reitsma JB, et al. Calculating the sample size required for developing a clinical prediction model. Bmj-Brit Med J. 2020;368. doi: 10.1136/bmj.m441.
43. de Mutsert R, Jager KJ, Zoccali C, Dekker FW. The effect of joint exposures: examining the presence of interaction. Kidney Int. 2009;75(7):677-81. doi: 10.1038/ki.2008.645.

44. Altman DG, Royston P. Statistics notes. The cost of dichotomising continuous variables. Brit Med J. 2006;332(7549):1080-. doi: 10.1136/bmj.332.7549.1080.
45. Lever J, Krzywinski M, Altman N. Regularization. Nature Methods. 2016;13(10):803-4. doi: 10.1038/nmeth.4014.
